# Health State Preference Weights for the Glasgow Outcome Scale Following Traumatic Brain Injury: A Systematic Review and Mapping Study

**DOI:** 10.1016/j.jval.2016.09.2398

**Published:** 2017-01

**Authors:** Gordon Ward Fuller, Monica Hernandez, David Pallot, Fiona Lecky, Mathew Stevenson, Belinda Gabbe

**Affiliations:** 1 Emergency Medicine Research in Sheffield, Health Services Research Section, School of Health and Related Research (ScHARR), University of Sheffield, Sheffield, South Yorkshire, UK; 2 School of Health and Related Research, University of Sheffield, Sheffield, South Yorkshire, UK; 3 Emergency and Trauma Research Unit, Department of Epidemiology and Preventive Medicine, Level 6, the Alfred Centre, Alfred Hospital, Melbourne, Victoria, Australia

**Keywords:** craniocerebral trauma, decision analysis models, economic models, EuroQol-5D, Glasgow Outcome Scale, health status indicators, mapping study, quality-adjusted life-years, quality of life, systematic review

## Abstract

**Background:**

Valid and relevant estimates of health state preference weights (HSPWs) for Glasgow Outcome Scale (GOS) categories are a key input of economic models evaluating treatments for traumatic brain injury (TBI).

**Objectives:**

To characterize existing HSPW estimates, and model the EuroQol five-dimensional questionnaire (EQ-5D) from the GOS, to inform parameterization of future economic models.

**Methods:**

A systematic review of HSPWs for GOS categories following TBI was conducted using a highly sensitive search strategy implemented in an extensive range of information sources between 1975 and 2016. A cross-sectional mapping study of GOS health states onto the three-level EQ-5D UK tariff index values was also performed in patients with significant TBI (head region Abbreviated Injury Scale score ≥3) from the Victoria State Trauma Registry. A limited dependent variable mixture model was used to estimate the 12-month EQ-5D UK value set as a function of GOS category, age, and other explanatory variables.

**Results:**

Six unique HSPWs from five eligible studies were identified. All studies were at high risk of bias with limited applicability. The magnitude of HSPWs differed significantly between studies. Three class mixture models demonstrated excellent goodness of fit to the observed Victoria State Trauma Registry data. GOS category, age at injury, sex, comorbidity, and major extracranial injury all had significant independent effects on mean EQ-5D utility values.

**Conclusions:**

The few available HSPWs for GOS categories are challenged by potential biases and restricted generalizability. Mixture models are presented to provide HSPWs for GOS categories consistent with the National Institute for Health and Care Excellence reference case.

## Introduction

Increasing health care demands, limited by finite health budgets, have necessitated economic evaluations of new health technologies to ensure efficient use of scarce resources [1]. Cost-effectiveness is commonly assessed by comparing interventions in terms of costs and quality-adjusted life-years, comprising duration of life expectancy weighted by preferences for health-related quality of life (HRQOL) over this period [2].

Incremental costs per quality-adjusted life-year in economic evaluations are often derived using decision analysis modeling to synthesize available evidence and represent hypothetical patients’ responses to alternative interventions [3]. Valid and relevant estimates of preference weights for defined outcome states (health state preference weights [HSPWs]) are a key input of such models [4]. Systematic literature searching is the methodological standard to ensure transparent and complete identification of HSPW estimates [5]. In the absence of valid empirical values for HSPWs, “mapping” of non–preference-based measures of health to generic-based measures can be performed [5], with the UK National Institute for Health and Care Excellence (NICE) recommending that HRQOL be measured directly from patients using the EuroQol five-dimensional questionnaire (EQ-5D) and valued by a UK tariff of general population preferences [6]. Background information on the EQ-5D is provided in the Web Appendix in Supplemental Materials found at http://dx.doi.org/10.1016/j.jval.2016.09.2398.

Traumatic brain injury (TBI) is a major public health problem in the United Kingdom, being one of the leading causes of death and disability and costing the economy an estimated £4 billion per year [7], [8]. Outcome in TBI effectiveness studies is conventionally assessed using the basic or extended Glasgow Outcome Scale (GOS), measuring death and severity of disability using an ordinal scale [9]. Health state valuations corresponding to these outcomes are therefore necessary for health economic models examining new health technologies in TBI, but despite their importance there is a paucity of suitable estimates.

The aim of this study was to provide a comprehensive reference source of HSPWs for GOS health states following adult TBI to inform parameterization of future economic models. Specific objectives were to systematically identify all HSPWs available at present for GOS states following TBI and to validly derive estimates of HSPWs for GOS from the EQ-5D.

## Methods

A systematic review and a cross-sectional study mapping GOS health states onto the three-level EQ-5D (EQ-5D-3L) UK tariff index values were conducted.

### Systematic Review

#### Study design and criteria for considering studies

A detailed review protocol stating an a priori analysis plan was developed before data collection. The review inclusion and exclusion criteria are presented in Table 1. All methods of obtaining HSPWs were eligible, with the exception of elicitation using expert opinion, which is limited by high risk of bias and lack of descriptive validity [5].

#### Identification of evidence

An extensive range of electronic information sources were examined including all major bibliographic databases, specialist health economic and gray literature databases, and relevant Web sites. Additional information sources included forward and backward citation searching, author searching, reference checking, and contact with experts. Search strategies for bibliographic databases were developed iteratively in conjunction with an information services specialist and underwent external peer review. Searches were conducted for original research published between 1975 (corresponding to the introduction of the GOS) and week 33, April 2015. Present awareness searches were conducted in MEDLINE and Embase (week 3, April 2016) immediately before submission. Full details on information sources and search strategies are presented in the Web Appendix in Supplemental Materials.

#### Selection of evidence and data extraction

All studies identified during searches were assessed in a three-stage process with an initial screening of titles for relevance, followed by further examination of abstracts and full-text articles as required to assess eligibility. Studies were then classified as follows: eligible if inclusion criteria were met; potentially eligible if information was collected that could potentially allow calculation of HSPWs but estimates were not reported (e.g., short form 36 health survey and GOS both measured simultaneously); or ineligible. Relevant information on study characteristics and methodology was then collected for eligible and potentially eligible studies using a prespecified data extraction form. Study selection and data extraction were performed by a single reviewer and checked by a second independent reviewer.

#### Appraisal of quality, data synthesis, and statistical analyses

Included HSPW studies were assessed for quality using a peer-reviewed critical appraisal checklist based on the NICE Decision Support Unit guidelines [5], the Cochrane risk of bias tool [10], and theoretical considerations (further details are provided in the Web Appendix in Supplemental Materials) [11], [12]. The risk of bias in each domain was subsequently rated as high, low, or unclear. A narrative synthesis of identified HSPWs was prespecified in the event that clinically and methodologically homogeneous studies at low risk of bias were not identified. To facilitate comparisons, reported measures of variance for HSPWs were converted to 95% confidence intervals (CIs). Extended GOS category HSPWs were combined using weighted averages to provide results for commensurate basic GOS health states. One-way analyses of variance (ANOVAs) using published summary statistics were used to test for statistically significant differences between HSPW estimates within each basic GOS category. Post hoc Scheffe multiple-comparison hypothesis tests for differences in means were then used to identify which HSPW estimates differed [13].

### Mapping Study

#### Study design

A retrospective cohort study was performed by analyzing data from the Victorian State Trauma Registry (VSTR) [14]. A model for predicting mean EQ-5D HSPWs for GOS categories at 12 months postinjury was developed using adjusted limited dependent variable mixture modeling. This model has consistently been shown to outperform other models when properly specified [15], [16], [17], [18], [19], and follows the draft of the International Society for Pharmacoeconomics and Outcomes Research Good Practices on mapping of utilities [20].

#### Setting and study population

The VSTR is a population-based database that has collected information on all major trauma cases within the state of Victoria, Australia, since 2001 [14]. Patients are included if they meet any of the following criteria: Injury Severity Score of more than 12; admission to critical care for more than 24 hours, with mechanical ventilation for at least part of that time, as a result of injury; urgent surgery secondary to major trauma; or death due to injury. Consecutive cases are prospectively identified from emergency admission data, discharge data, review of hospital case notes, and coroners’ records by VSTR data collectors in each hospital. The study population comprised consecutive adults (≥16 years) enrolled in VSTR with significant TBI (head region Abbreviated Injury Scale severity score ≥3) and injured between January 2008 and June 2013 [21], [22]. Deceased patients in the GOS 1 category by definition had an HSPW value of 0 and were excluded from consideration in predictive models.

#### Data collection

The VSTR contains an extensive data set of demographic, physiological, injury, investigation, and treatment and outcome variables. Data are collected from prehospital and inpatient case notes, hospital information systems, and the National Coroner’s Information System and submitted by electronic upload or Web-based entry systems. Linkage between separate hospital admissions in the case of interhospital transfer is achieved deterministically on the basis of demographic identifiers. Postdischarge follow-up is conducted by telephone interviews at 6, 12, and 24 months and includes standardized questionnaires for the extended GOS and the EQ-5D-3L. To meet the NICE reference case for economic evaluations, the EQ-5D-3L was then valued using the UK tariff [6], [23].

#### Statistical analyses

The injury and demographic features of the study cohort were characterized using descriptive statistics. Adjusted limited dependent variable mixture models were then developed to predict mean EQ-5D preference weights for each basic GOS health state at 12 months, conditional on important patient characteristics [17], [19], [24]. An initial simple model was developed with 12-month EQ-5D as the dependent variable and GOS category and age as explanatory variables. An additional detailed model was developed, with age, sex, comorbidities, and the presence of extracranial injury evaluated as further covariates likely to be important characteristics of TBI populations modeled in economic evaluations. The goodness of model fit was evaluated using information criterion statistics, mean absolute error, root mean squared error, and visual comparison of predicted and observed values. Models were validated by out-of-sample predictions for EQ-5D at 6 and 24 months postinjury within the same cohort, and comparing cumulative distribution functions. Secondary analyses examining the extended GOS and a range of international EQ-5D tariff values were also performed. Further details on adjusted limited dependent variable mixture models, the modeling strategy, and secondary analyses are given in the Web Appendix in Supplemental Materials.

#### Ethics, funding, and statistical software

The VSTR has the approval of the Human Research Ethics Committee to collect data from all participating health services. Specific ethical approval for the present study was obtained from the Monash University Human Research Ethics Committee, and the Steering Committee of the VSTR approved the provision of de-identified data for this study. A two-sided *P* value of less than 0.05 was considered to be statistically significant. All statistical analyses were carried out in Stata version 12.1 (StataCorp, College Station, TX). The Stata aldvmm module was used to perform adjusted limited dependent variable mixture modeling [24].

## Results

### Systematic Review

#### Study selection

**Fig. 1:**
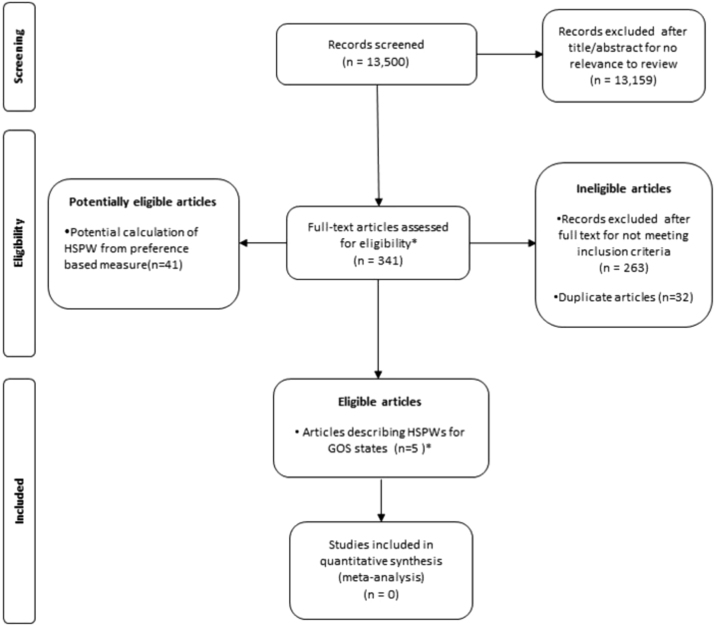
PRISMA flowchart describing systematic review study selection. De-duplication was performed at the full-text stage and a one-to-one relationship subsequently existed between articles and studies. *Six unique GOS HSPWs reported in five articles. GOS, Glasgow Outcome Scale; HSPWs, health state preference weights; PRISMA, Preferred Reporting Items for Systematic Reviews and Meta-Analyses.

In this study, 13,500 citations were screened for eligibility, with the full text of 341 articles retrieved for detailed evaluation. During full-text examination, five studies were found that described six sets of HSPWs for GOS or comparable health states following TBI [18], [19], [20], [21]. Forty-one potentially eligible “near-miss” studies were collected on GOS and a preference-based health state description scale (or a non–preference-based HRQOL instrument with established mapping function) but were ultimately not included because HSPW estimates were not reported. Figure 1 shows the selection of studies in detail.

#### HSPW study characteristics

Two studies used case scenarios to describe health states corresponding to GOS categories ([40] [extended GOS]; Aoki, 1995 [GOS]). Preferences for HRQOL were then directly determined by external populations using the standard gamble technique [12]. A third study (Djikers, 2004) also used case vignettes, broadly comparable to GOS states, to formulate HSPWs. Quality of Well-Being and Health Utility Index 3 generic multiattribute health description instruments were then applied by the author, allowing indirect determination of preferences using the appropriate preference valuation algorithm [12]. [42] measured GOS and HRQOL using the Rosser Index of Health-Related Quality of Life [12], asking patients to recall their health status in the years following their head trauma. Preferences were then determined indirectly for GOS categories using the Rosser valuation matrix, and a smoothing regression function was applied to estimate mean utility for each year from 1 to 7 postinjury. Finally, [43] measured both the GOS and the EQ-5D in a sample of patients with complicated mild head injury. Preferences were then determined indirectly for the GOS categories from the Dutch EQ-5D tariff [12]. The characteristics of included HSPW studies are presented in further detail in the Web Appendix in Supplemental Materials.

#### Reported results and risk of bias

HSPW estimates for each GOS state are presented in Table 2. Significant variations in HSPWs were evident, with those reported by [42] being appreciably higher across all GOS categories (ANOVA *P* < 0.001). Despite the relatively small sample sizes, these differences reached statistical significance for categories of moderate and severe disability (ANOVA and Scheffe multiple-comparison test *P* < 0.001). Differences were in excess of previously reported minimum clinically important differences for the EQ-5D (mean 0.074 [range −0.011 to 0.140]) [25].

Risk of bias was high for each study using health state scenarios ([40]; Djikers, 2004; Aoki, 1995) secondary to unvalidated GOS descriptions and nonrepresentative valuing populations. Risk of bias was also high for studies measuring health states directly from patients. Additional information available from [43] indicated substantial loss to follow-up (43%). [42] also reported marked loss to follow-up (72%), and was further limited by the reliance on patients remembering their health status from several years previously, with the consequent potential for recall bias. The risk of bias for each HSPW estimate is presented in Table 3, with a detailed rationale presented in the Web Appendix in Supplemental Materials.

### Mapping of GOS Categories onto UK Tariff EQ-5D Index Values

#### Sample characteristics

**Fig. 2:**
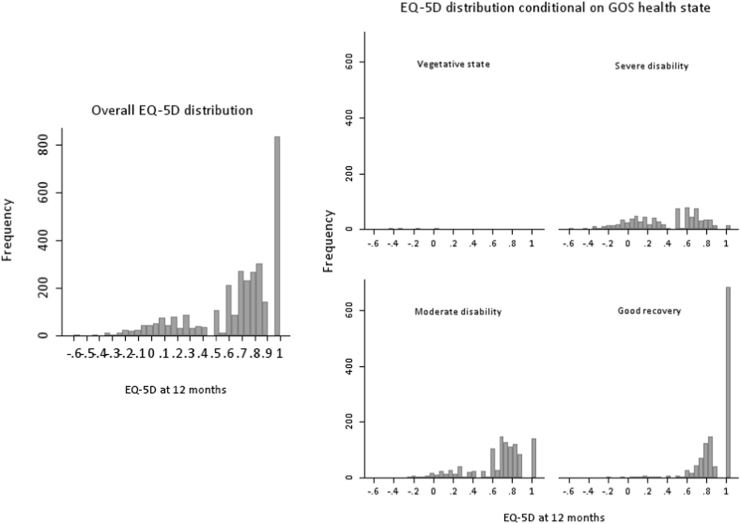
Distribution of EQ-5D at 12 months overall, and stratified by basic GOS category. EQ-5D, EuroQol five-dimensional questionnaire; GOS, Glasgow Outcome Scale.

A study sample of 3437 VSTR patients meeting inclusion criteria and with complete information on 12-month EQ-5D and 12-month GOS was included in a complete case analysis. The median age of the study sample was 50 years (interquartile range 29–72), with males accounting for 71.3% of cases (95% CI 69.8–72.9%). The median Injury Severity Score was 21 (interquartile range 16–26). The distributions of 12-month EQ-5D values, overall and after stratification by GOS category, are presented in frequency histograms in Figure 2. Typical features of the EQ-5D distribution including multimodality, local maxima with variable skewness and kurtosis, discontinuity, and distinct probability masses were evident [12]. Further details on derivation of the study sample and patient characteristics are provided in the Web Appendix in Supplemental Materials. Briefly, there was a moderate proportion of missing data for important study variables, ranging from 0% for age and sex to 27% for 12-month EQ-5D. Patients excluded from the available case analyses because of missing data had characteristics similar to those of included patients.

#### Predictive modeling of EQ-5D preference weights on the basis of GOS category

**Fig. 3:**
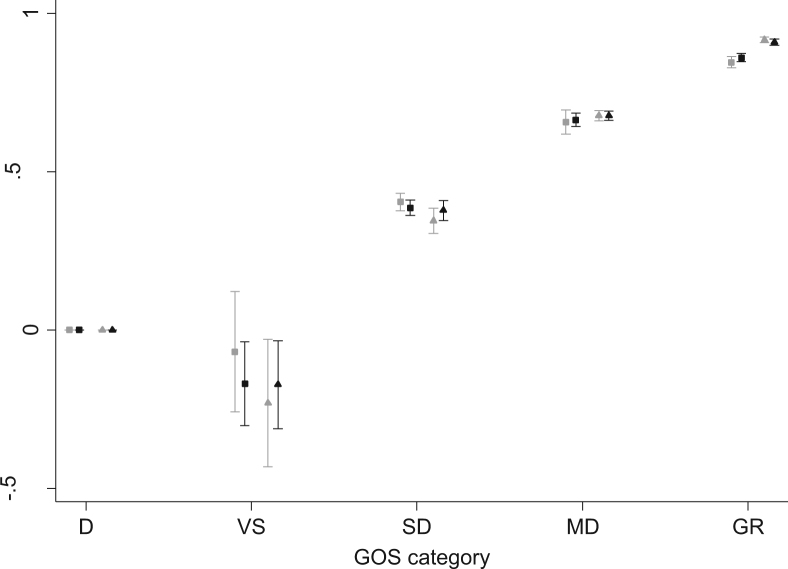
Mean predicted 12-month EQ-5D value for each basic GOS category at representative ages compared with observed mean values. Black symbols represent point estimates from the simple adjusted limited dependent variable mixture model for predicted mean EQ-5D values conditional on age (▲, <65 years old, ■, ≥65 years old) and basic GOS category (D, dead; VS, vegetative state; SD, severe disability; MD, moderate disability; GR, good recovery). Gray symbols represent corresponding mean observed EQ-5D values. Error bars report 95% CI for observed and predicted mean. CI, confidence interval; EQ-5D, EuroQol five-dimensional questionnaire; GOS, Glasgow Outcome Scale.

**Fig. 4:**
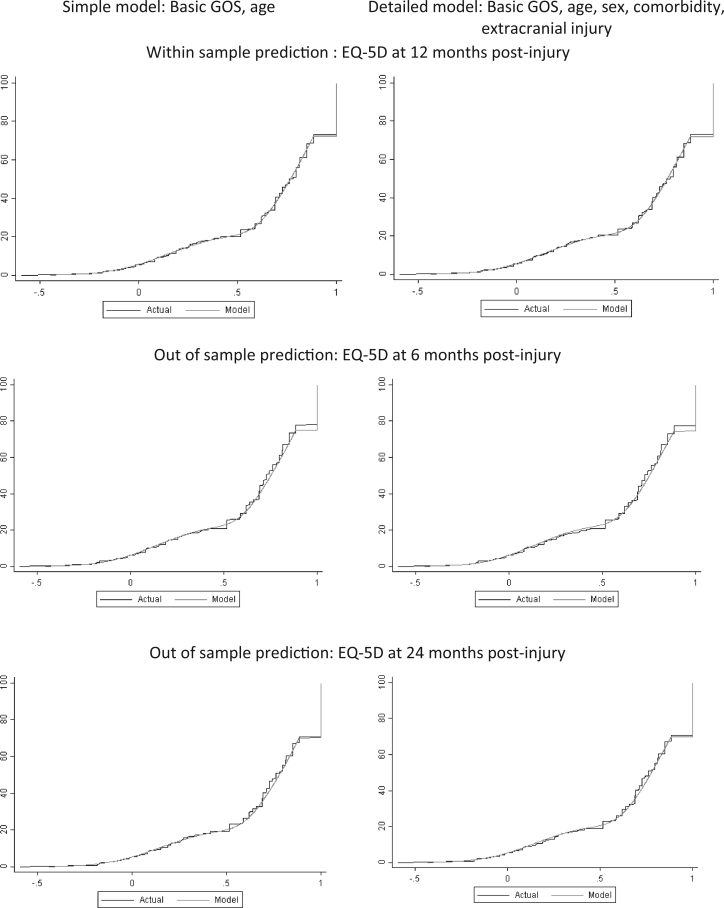
Cumulative distribution functions for observed vs. predicted EQ-5D values from predictive models. The *x*-axis denotes the EQ-5D and the *y*-axis displays cumulative percentage of cases. EQ-5D, EuroQol five-dimensional questionnaire; GOS, Glasgow Outcome Scale.

The preferred model predicting EQ-5D index scores from GOS category and age, chosen on the basis of parsimony and relative favorability of goodness-of-fit statistics (Table 4), included three latent classes. Model coefficients are presented in Table 5. The addition of a fourth latent class provided a negligible improvement in model fit at the expense of increased model complexity. Age and GOS category demonstrated a significant effect on the probability of latent class membership and the distribution of mean EQ-5D scores within each component. As shown in Figure 3, model predictions showed excellent concordance with observed values, demonstrating relatively lower HSPWs for vegetative state and severe disability GOS categories in younger patients than in older patients, with the opposite relationship apparent for favorable GOS categories of moderate disability and good recovery. Out-of-sample prediction of EQ-5D at 24 months postinjury also showed excellent agreement between observed and predicted values as shown by the cumulative distribution functions in Figure 4. There was, however, an underprediction of the EQ-5D at 6 months for those patients with little or no functional disability (Fig. 4).

The most favorable detailed model, including the additional covariates of age, sex, comorbidity, and extracranial injury, also used three latent classes (Table 5). Each covariate had a significant association with the probability of latent class membership, or the EQ-5D distribution within each component. A similar pattern of excellent in-sample (data not shown) and out-of-sample prediction at 24 months postinjury was evident but underprediction of high EQ-5Ds at 6 months postinjury was again observed (Fig. 3). Further details on the results of the detailed model for GOS, including variable coefficients, are provided in the Web Appendix in Supplemental Materials.

Results of secondary analyses examining international tariff EQ-5D estimates for GOS categories, and adjusted limited dependent variable mixture models including extended GOS categories, are presented in the Web Appendix in Supplemental Materials. Briefly, the EQ-5D estimates varied significantly between countries both statistically (Friedman test *P* < 0.001) and clinically (differences in excess of minimum clinically important differences). Preferred models predicting EQ-5D index scores from extended GOS category included four latent classes, with each covariate having a statistically significant association with the EQ-5D distribution within each component. A Stata “do file” providing mean predicted EQ-5D and 95% CIs, conditional on GOS category and covariates, is provided as an additional supplementary file.

## Discussion

### Summary of Results

The few existing estimates for HSPWs of GOS categories are at high risk of bias and demonstrate significant variation in values. Adjusted limited dependent variable mixture models are presented providing predictions for the EQ-5D–based HSPWs of GOS and extended GOS categories meeting the UK NICE reference case. In addition to GOS category, age at injury (simple model) and age, sex, comorbidity, and major extracranial injury (detailed model) all have small but significant independent effects on mean EQ-5D utility values. Predictions from these models demonstrated excellent goodness of fit to the observed data.

### Interpretation of Findings

There is a large empirical evidence base demonstrating that the methods and populations used for health state measurement and valuation will influence the magnitude of resulting HSPWs [12], [26], [27], [28], [29], [30], [31], [32]. Given that the eligible HSPWs identified in the systematic review comprised a heterogeneous range of study designs, it is therefore unsurprising that GOS estimates differed significantly between studies. Djikers (2004) used an unorthodox method of describing health states with subsequent application of generic preference-based health measures. The lack of descriptive validity inherent in this approach prevents meaningful interpretation of results. The Rosser Index of Health-Related Quality of Life has not been widely studied, and it is therefore difficult to comment on the comparability of utility values reported by Tsauo (1999) [33]. The finding that [43], who used the EQ-5D instrument to measure health status from patients with mild TBI, reported the lowest HSPWs of the remaining studies is consistent with the previous observation that indirect valuation tends to result in lower health ratings compared with direct methods [12]. [40] and [41] directly valued scenarios with the standard gamble approach, and a number of factors may have been important in producing the disparate results reported. First, Aoki used very brief GOS category descriptions, compared with Kosty’s more detailed narrative vignettes, which may have led respondents to use their own judgments on the impact of each state with consequently lower valuations. Second, labeling of health states as posthead injury by Kosty could have resulted in framing effects, although this would generally be expected to lower HSPW values [26]. Third, evidence suggests that preferences over health states can be constructed during interviews, and differences in the format of the standard gamble exercises may have been important [34]. Finally, little is known about the impact arising from valuation of health states by health professionals compared with the general public.

In addition to poor internal validity, it is notable that the studies identified in this systematic review are likely to have limited relevance in future TBI models, underlining the importance of the developed predictive models. International cost-utility guidelines generally mandate measurement of health from patients with standardized and validated generic HRQOL preference measures [6], [12], [35], limiting the applicability of Djikers (2004), [40], and [41] estimates. Additional concerns are the valuation of preferences by health professionals in Aoki’s study and the nonrepresentative general population sample providing preferences in Kosty’s study. Although [43] used the EQ-5D instrument to measure HRQOL from patients, the sample population comprised a narrow subset of patients with mild head injury with intracranial CT abnormalities that may have limited generalizability to other TBI subgroups. Little information is available on the methodology used to derive the Rosser health classification system’s valuation matrix and the applicability is consequently uncertain.

Interestingly, the VSTR predictive model suggests that mean EQ-5D value for unfavorable GOS categories of vegetative state and severe disability could be lower in younger patients. Such a pattern could arise if respondents, or their proxies, perceive a given level of disability to have a lesser impact at older age. Nevertheless, because this finding did not reach statistical significance, the pattern observed may represent the play of chance. The opposite relationship between age and utility was apparent for favorable outcomes of moderate disability and good recovery, in which utility was significantly less in the oldest age groups compared with the youngest patients. This finding is unsurprising because declining HRQOL with age is well established secondary to increasing prevalence of comorbidities and infirmity [36]. Supporting this position, the influence of age on utility was reduced in the more detailed predictive model once comorbidity was added as an explanatory variable. The reduction in utility associated with major extracranial injury seen in this model is also intuitive because HRQOL would be expected to decrease in the presence of pain, depression, and anxiety caused by nonhead injuries, but which are not fully assessed in the GOS. In common with previous studies in other disease areas, a reduction in utility associated with females was apparent [37], [38].

A further notable result was that models predicting 12-month EQ-5D values performed well at 24 months, but underpredicted EQ-5D at 6 months for those patients with little or no functional disability. This finding is likely explained by the evolving nature of functional recovery during the early postinjury stage, which may have a bigger impact on EQ-5D within the top categories of GOS. This position is supported by findings from the Traumatic Brain Injury Model Systems National Database which found that functional status initially improves rapidly before plateauing [39].

The VSTR predictive model is consistent with the NICE reference case and should therefore be directly relevant to future UK health technology appraisals [6]. Nevertheless, for these results to be applicable to other jurisdictions, the statistical relationship between GOS categories and mean EQ-5D value must be the same in the economic evaluation’s modeled population as that in the VSTR estimation sample. Differences could arise because of the type of TBI, variation in population attributes, or timing of assessment. Overall, given the similar economic and demographic characteristics, the results are likely to be applicable to North American and Western European populations in the first few years following TBI, but generalizability to other countries and time points is less certain.

### Study Strengths and Limitations

The systematic review benefits from concordance with methodological guidelines, sensitive search terms, and an extensive search strategy covering all potentially useful information sources. Identifying HSPW data, however, is challenging because of the lack of validated methodological search filters, nonspecific thesauri terms in bibliographic databases, and lack of clear reporting of HSPW studies in titles and abstracts [5]. It is therefore possible that not all GOS HSPWs were identified. There are no accepted critical appraisal tools for judging the risk of bias or evaluating the use of HSPWs within decision analysis models, and although we have based our assessment on published recommendations and expert guidelines our methods have not been fully validated.

The utility mapping study also has a number of strengths. Because data submission to VSTR is mandatory, incomplete database enrollment is unlikely and the study’s sampling frame can be considered to be comprehensively population-based. Levels of missing covariate data were comparatively low because of careful matching of interhospital transfers and thorough data collection processes. Furthermore, the mixture modeling approach is specifically designed to produce appropriate estimates of EQ-5D from clinical and other predictors, and has been shown to be superior to other statistical techniques.

Conversely, a number of limitations arising from patient attrition, sparse data, measurement error, and uncertainties surrounding EQ-5D index values could potentially undermine the internal validity of results. Although the relatively high loss to follow-up of 20% raises the possibility of selection bias, included cases appeared to be representative of the overall study population (see the Web Appendix in Supplemental Materials). Very few patients (six cases) with vegetative state were available in the study sample, leading to imprecise point estimates and very large standard errors for relevant coefficients in the primary model examining basic GOS and age. Nevertheless, repeating the analyses after combining vegetative state and severe disability categories, or excluding vegetative state patients from the estimation sample, did not change predictions for other health states, suggesting that sparse data for this outcome group did not adversely affect model performance (data not shown). Systematic misclassification of GOS categories and other covariates, or differential measurement error in EQ-5D assessments, could lead to incorrect HSPW estimates. Random errors arising from interobserver variability or coding mistakes are, however, more plausible, leading to unbiased but less precise predictions. An additional limitation is that the predictive models have assumed that EQ-5D index values are actual distinct values, when in reality they are themselves uncertain estimates derived from a regression model [23]. The 95% CIs surrounding the reported HSPWs are consequently slightly underestimated.

## Conclusions

This is the first known study to systematically identify, characterize, and appraise available HSPWs for GOS categories following TBI. The small number of existing estimates are challenged by potential biases and restricted generalizability, limiting their use in TBI economic evaluations. We have consequently developed a robust model giving valid and applicable utility estimates for GOS health states meeting the NICE reference case, providing a valuable resource for parameterization of future economic models. A program file is supplied, allowing simple calculation of mean utility values and 95% CIs conditional on important patient characteristics.

## Figures and Tables

**Table 1:** Review inclusion and exclusion criteria

*Inclusion criteria*
• Disease: Mild, moderate, or severe TBI* • Disease population: Adult patients with TBI > 16 y • Health states: Consistent with GOS categories • Population describing health states: Patients, carers, or health professionals • Method for measuring HRQOL for each health state: Scenarios, generic multiattribute utility instruments, disease-specific multiattribute health instruments, direct measurement • Method for determining preferences for HRQOL for each health state: Direct preference-based valuation of GOS health states using recognized elicitation method (SG, VAS, or TTO); indirect valuation of GOS health states (following health state measurement with generic or disease-specific multiattribute utility instruments) using recognized elicitation method (SG, VAS, or TTO). • Population providing preferences for HRQOL of health states: General public, patients, carers, health professionals • Study types: Original HSPV research study reporting at least one unique HSPV • Language: English language or available translation • Dates: 1975 to present
*Exclusion criteria*
• Disease: Non-TBI conditions (e.g., stroke) • Disease population: Pediatric patients, aged < 16 y • Health state measurement: Methods not suitable for mapping to the EQ-5D • Health state valuation method: Non–preference-based valuation of health states (e.g., expert opinion used to value health states)

AIS, Abbreviated Injury Scale; EQ-5D, EuroQol five-dimensional questionnaire; GCS, Glasgow Coma Scale; GOS, Glasgow Outcome Scale; HRQOL, health-related quality of life; HSPW, Health State Preference Weights; SG, standard gamble; TBI, traumatic brain injury. TTO, time trade-off; VAS, visual analogue scale.

⁎ TBI severity categorized according to GCS score or head region AIS score: mild TBI: GCS 14–15 or AIS 1 or 2; moderate TBI: GCS 9–13 or AIS 3; severe TBI: GCS ≤ 8 or AIS 4–6.

**Table 2:** HSPW estimates for basic and extended GOS categories

**GOS category**		**Utility estimates: mean (95% CI)**						**ANOVA for basic GOS states**¶
	[41]*	[42]†, ‡	**Djikers (2004)**†, §**: QWB**	**Djikers (2004)**†, §**: HUI3**	[43]*	[40]: GOS||	[40]: GOSE	
	**(n = 140)**	**(n = 99)**	**(n = 1)**	**(n = 1)**	**(n = 87)**	**(n = 101)**	**(n = 101)**	

1: Death	0.0 (0.0)	0.0	0.0	0.0	0.0 (0.0–0.0)	0.0 (0.0)	0.0 (0.0)	–
2: Persistent vegetative state*	0.08 (0.05–0.11)	–	–	–	–	0.11 (0.07–0.15)	0.11 (0.07–0.15)	*P* = 0.24
3: Severe disability	0.26 (0.22–0.30)	0.71	0.43	0.13	0.15 (0.06–0.28)	0.50 (0.46–0.53)	–	*P* < 0.001
							0.41 (0.37–0.45)	
[GOSE 3: Lower severe disability]								
							0.58 (0.54–0.62)	
[GOSE 4: Upper severe disability]								
4: Moderate disability	0.63 (0.58–0.68)	0.94	0.53	0.48	0.51 (0.39–0.63)	0.76 (0.73–0.78)	–	*P* < 0.001
			0.47	0.33			0.70 (0.67–0.73)	
			0.60	0.63				
							0.81 (0.78–0.84)	
[GOSE 5: Lower moderate disability]								
[GOSE 6: Upper moderate disability]								
5: Good recovery	0.85 (0.82–0.88)	0.94	0.80	0.93	0.88 (0.71–0.97)	0.93 (0.91–0.95)	–	*P* = 0.24
							0.86 (0.83–0.89)	
[GOSE 5: Lower good recovery]								
							1.00 (1.00–1.00)	
[GOSE 6: Upper good recovery]								

ANOVA, analysis of variance; CI, confidence interval; GOS, Glasgow Outcome Scale; GOSE, extended GOS; HSPW, health state preference weight; HUI, Health Utility Index; NA, not applicable; QWB, Quality of Well-Being.

⁎ Persistent vegetative state not observed in [42]/[43] and not assessed for [44].

† Variance not reported in [42] and not applicable for [44].

‡ [42] reported multiple HSPW estimates across several years of follow-up for each GOS category. The category-specific mean HSPW is reported herein.

§ Indirect determination of preferences for health states measured from scenarios using generic multiattribute preference-based health description instrument.

|| Basic GOS categories calculated from published GOSE scores using weighted averages.

¶ ANOVA for basic GOS categories only.

**Table 3:** Critical appraisal of HSPW estimates: Risk of bias in each domain rated high, low, or unclear

**Study**	**Health state description and measurement**		**Health state valuation**		**Other sources of bias**	**Overall**
	**Selection bias**	**Information bias**	**Selection bias**	**Information bias**		
[40]	NA	Low	High	Low	Low	High
[43]	High	Low	Low	Low	Low	High
Djikers (2004)	NA	High	High	Low	Low	High
[42]	High	High	Unclear	Unclear	Low	High
[41]	NA	High	Unclear	Low	Low	High

HSPW, health state preference weight; NA, not applicable.

**Table 4:** Goodness-of-fit metrics for simple and detailed models predicting EQ-5D from basic GOS category

**Number of latent classes**	**AIC**	**BIC**	**Mean error**	**MAE**	**RMSE**
*Simple model: Basic GOS and age*					
1	2173.715	2210.569	0.006147	0.196555	0.25746045
2	1023.686	1128.106	−0.00076	0.193722	0.25608219
3	960.9117	**1120.613**	**−0.000605**	0.193344	0.25592006
4	**955.7214**	1170.704	−0.000645	**0.193304**	**0.25589877**

Number of latent classes	AIC	BIC	Mean error	Absolute error	RMSE
*Detailed model: Basic GOS and age, sex, comorbidity, extracranial injury*					
1	869.4129	**1026.339**	**−0.00067**	0.194383	0.25614
2	820.5065	1031.753	−0.00077	0.192999	0.254911
3	**806.3804**	1029.698	−0.00076	**0.192481**	**0.25466**
4	810.1862	1045.575	−0.00079	0.192521	0.254685

*Note*. Most favorable result for each metric of goodness of fit highlighted in bold text.

AIC, Akaike information criterion; BIC, Bayesian information criterion; EQ-5D, EuroQol five-dimensional questionnaire; GOS, Glasgow Outcome Scale; MAE, mean absolute error; RMSE, root mean squared error.

**Table 5:** Coefficients for the initial adjusted limited dependent variable mixture model predicting 12-mo EQ-5D HSPWs from basic GOS category and age

Variable*	**Coefficient**	**SE**	***P*****value**		**95% CI**
*Explanatory variables within component 1*					
Vegetative state	−0.524	0.077	0.000		−0.675 to −0.373
Severe disability	−0.196	0.049	0.000		−0.293 to 0.099
Moderate disability	−0.053	0.051	0.296		−0.154 to 0.047
Age	0.001	0.005	0.815		−0.008 to 0.010
Constant	0.280	0.053	0.000		0.176 to 0.385
*Explanatory variables within component 2*					
Vegetative state	−0.778	0.114	0.000		−1.002 to −0.554
Severe disability	−0.001	0.092	0.991		−0.182 to 0.180
Moderate disability	0.041	0.077	0.598		−0.111 to 0.192
Age	−0.011	0.002	0.000	−0.015 to −0.008	
Constant	0.844	0.054	0.000		0.737 to 0.950
*Explanatory variables within component 3*					
Vegetative state†	–	–	–	–	–
Severe disability	−0.337	0.047	0.000		−0.429 to −0.245
Moderate disability	−0.282	0.036	0.000		−0.353 to −0.211
Age	−0.012	0.002	0.000		−0.015 to −0.009
Constant	1.002	0.020	0.000		0.962 to 1.042
*Explanatory variables explaining the probability of component 1 membership*					
Vegetative state	20.562	1.441	0.000		17.738 to 23.386
Severe disability	3.962	0.298	0.000		3.378 to 4.546
Moderate disability	2.785	0.333	0.000		2.133 to 3.437
Age	0.002	0.039	0.957		−0.075 to 0.079
Constant	−3.143	0.273	0.000		−3.679 to −2.607
*Explanatory variables explaining the probability of component 2 membership*					
Vegetative state	17.000	–	–	–	–
Severe disability	0.697	0.244	0.004		0.219 to 1.174
Moderate disability	1.285	0.234	0.000		0.827 to 1.744
Age	0.077	0.033	0.021		0.012 to 0.142
Constant	−1.029	0.736	0.162		−2.471 to 0.413
Sigma					
Sigma 1	0.212	0.009			0.195 to 0.230
Sigma 2	0.086	0.019			0.056 to 0.131
Sigma 3	0.061	0.013			0.041 to 0.092

CI, confidence interval; EQ-5D, EuroQol five-dimensional questionnaire; GOS, Glasgow Outcome Scale; HSPWs, health state preference weights; SE, standard error.

⁎ Basic GOS coded as indicator variable with GOS 5 (good recovery) as the baseline category. Basic GOS category 1 (death) not modeled because this will equal 0 by definition.

† There was a zero probability of membership of class 3 if in persistent vegetative state. This coefficient was therefore constrained to 0. Covariance matrix is available on request. A Stata “do file” allowing calculation of mean EQ-5D value with 95% CIs for a given basic GOS and age category is supplied as an additional file.
