# Supplementary material for: Health State Preference Weights for the Glasgow Outcome Scale Following Traumatic Brain Injury: A Systematic Review and Mapping Study
Source: Value Health. 2017 Jan;20(1):141–51. doi: 10.1016/j.jval.2016.09.2398 (PMC5339086; doi:10.1016/j.jval.2016.09.2398)
Supplement: Supplementary file 1 — Supplemental Materials [file mmc1.docx]

**HEALTH STATE PREFERENCE WEIGHTS FOR THE GLASGOW OUTCOME SCALE FOLLOWING TRAUMATIC BRAIN INJURY: A SYSTEMATIC REVIEW AND MAPPING STUDY**

**Web Appendix**

*Supplementary Methodological Information and Results*

**Supplementary Methodological Information**

BACGROUND INFORMATION: eq-5d

The NICE reference case has historically required that HRQOL is measured directly from patients using the 3 level version of the EQ5D and valued by a UK tariff of general population preferences.([1](#_ENREF_1)) This preference-based multi-attribute health description instrument assesses HRQOL in 5 dimensions: mobility; self-care, usual activities, pain/discomfort; and anxiety/depression. Each dimension has 3 possible levels: no problems; some problems; or extreme problems, giving a total of 243 possible health states. The descriptive system is generic and addresses salient features of HRQOL relevant to a wide spectrum of diseases and treatments.([2](#_ENREF_2)) Alternative methods are only accepted by NICE when it has been clearly demonstrated that the EQ5D instrument lacks content validity for the condition in question.([1](#_ENREF_1)) The EQ5D questionnaire can be self-completed or administered by researchers using face-to-face or telephone interviews.([3](#_ENREF_3))

Preferences are then applied to the described EQ5D health state using a scoring algorithm. For UK applications a valuation tariff is available based on a sample of 3,395 members of the general population.([4](#_ENREF_4)) Preferences for 43 EQ5D health states were obtained using the time trade off technique and a linear regression model developed to predict preferences for the full range of EQ5D health profiles. This model results in a default utility of 1 when there are no problems in any health dimension. There is a constant decrement for any kind of health problem, subtractions for moderate or severe problems in each domain, and an additional negative constant term (the ‘N3’ term) if there is any dimension with severe problems. Alternative tariffs have been developed for different socioeconomic classes and nationalities, resulting in different preference values. The UK EQ5D 3 level descriptive system and scoring algorithm are detailed in Table 1. More recently a 5 level version of the EQ-5D has been introduced.([5](#_ENREF_5))

**Table 1. EQ5D-3L classification and scoring algorithm(**[**6**](#_ENREF_6)**)**

| Dimension | Level | Description | Scoring algorithm |  |
| --- | --- | --- | --- | --- |
|  |  |  | Full health: | 1.00 |
|  |  |  | Any problem: | -0.081 |
|  |  |  | Any level 3 problem: | -0.269 |
| Mobility | 1 | No problems walking about |  |  |
|  | 2 | Some problems walking about |  | -0.069 |
|  | 3 | Confined to bed |  | -0.314 |
| Self care | 1 | No problems with self care |  |  |
|  | 2 | Some problems washing or dressing |  | -0.104 |
|  | 3 | Unable to wash or dress self |  | -0.214 |
| Usual activities | 1 | No problems performing usual activities |  |  |
|  | 2 | Some problems performing usual activities |  | -0.036 |
|  | 3 | Unable to perform usual activities |  | -0.094 |
| Pain/discomfort | 1 | No pain or discomfort |  |  |
|  | 2 | Moderate pain or discomfort |  | -0.386 |
|  | 3 | Extreme pain or discomfort |  | -0.123 |
| Anxiety/depression | 1 | Not anxious or depressed |  |  |
|  | 2 | Moderately anxious or depressed |  | -0.071 |
|  | 3 | Extremely anxious or depressed |  | -0.236 |

Perfect health with no problem in any EQ5D health dimension is scored as 1. The presence of any problem results in a constant reduction of 0.081. Level 2 or 3 problems in each dimension has a specific associated decrement. The presence of any level 3 problem results in a further one-off subtraction of 0.269.

systematic review: Information sources

*Electronic sources*

1. Cochrane library: Cochrane database of systematic reviews; Cochrane controlled trials register; NHS Economic Evaluation Database
2. PUBMED
3. MEDLINE
4. EMBASE
5. CINAHL
6. Cost-effectiveness Analysis Registry
7. Health Economics and Evaluation database (HEED)
8. Research Papers in Economics (RePEc)
9. Patient-reported outcome and quality of life instruments database (PROQOLID)
10. Conference Proceedings Citation Index - Science
11. BIOSIS preview
12. SIGLE
13. Index to UK Theses
14. ProQuest Dissertation & Theses Database
15. Health Technology Assessment Agency, National Institute of Clinical Excellence, MAPI websites
16. EQ-5D, SF-6D, HUI websites
17. QOLIBRI website
18. Science Citation Index (author and citation searching)

*Non-electronic information sources*

1. Checking reference lists of retrieved article
2. Checking reference lists of existing literature reviews
3. Correspondence with experts in the field, and relevant study authors

Systematic review: SEARCHES

**Methods:** Search strategies for bibliographic databases were developed iteratively in conjunction with an information services specialist, and underwent independent expert peer review (Royal Society of Medicine). Relevant published search filters were consulted to inform the initial search strategy which was subsequently modified in light of retrieved reports and studies identified for inclusion. The MEDLINE search is listed below and was adapted for use in other data sources. A current awareness search was conducted prior to submission of the systematic review. References were managed in EndNote (Thomson Reuters, CA, USA) and Excel (Microsoft, Redmond, USA).

**Review question:** What HSPWs estimates are available for Glasgow Outcome Scale health states following mild, moderate and severe TBI in adult patients?

**Platform:** Ovid MEDLINE(R) In-Process & Other Non-Indexed Citations and Ovid MEDLINE(R) 1946 to Present

**Date limits:** 1975 – Week 33, April 2015.

Current awareness searches conducted to week 3, April 2016, immediately prior to submission.

**Other limits:** Human only, no editorials/comments/letters

**Search terms:**

1. exp Craniocerebral Trauma/
2. ((cerebral or craniocerebral or intracranial or cranio-cerebral or intra-cranial or cranial or head or brain or neurological) adj trauma$).ti,ab.
3. ((cerebral or craniocerebral or intracranial or cranio-cerebral or intra-cerebral or cranial or head or brain or neurological) adj injur$).ti,ab.
4. (Traumatic adj ((cerebral or craniocerebral or intracranial or cranio-cerebral or intra-cranial or cranial or head or brain or neurological) and injur$)).ti,ab.
5. (neurotrauma or neuro-trauma).ti,ab
6. 1 or 2 or 3 or 4 or 5
7. quality of life/
8. value of life/
9. quality of life.ti,ab.
10. life quality.ti,ab.
11. (Hql or HRQL or QOL or HRQOL).ti,ab.
12. Quality-Adjusted Life Years/
13. quality adjusted life year$.ti,ab.
14. qaly$.ti,ab.
15. Disability adjusted life.ti,ab.
16. daly$. Ti,ab.
17. Health status indicators/
18. (sf 36 or sf36 or sf thirtysix or sf thirty six or short form 36 or short form thirty six or short form thirty-six or short form 36 or rand36 or rand 36 or rand-36).ti,ab.
19. (sf6 or sf 6 or short form 6 or shortform 6 or sf six or sfsix or short form six or short form six).ti,ab.
20. (sf12 or sf 12 or short form 12 or shortform 12 or sf twelve or sftwelve or shortform twelve or short form twelve).tw.
21. (sf16 or sf 16 or short form 16 or shortform 16 or sf sixteen or sfsixteen or shortfrom sixteen or short form sixteen).tw.
22. (sf20 or sf 20 or short form 20 or shortform 20 or sf twenty or sftwenty or shortform twenty or short form twenty).tw.
23. (euroqol or eq5d or eq 5d or eq-5d).ti,ab.
24. health$ year$ equivalent$.ti,ab.
25. hye$.ti,ab.
26. health utilit$.ti,ab.
27. (hui or hui1 or hui2 or hui3).ti,ab.
28. quality of wellbeing$.ti,ab.
29. quality of well being.ti,ab.
30. Qwb.ti,ab.
31. (qald$ or qale$ or qtime$).ti,ab.
32. standard gamble$.ti,ab.
33. time trade off.ti,ab.
34. time tradeoff.ti,ab.
35. visual analog$ scale$ or likert.ti,ab.
36. discrete choice experiment$.ti,ab.
37. (TTO or SG or VAS).ti,ab.
38. health state$ utilit$.ti,ab.
39. health state$ value$.ti,ab.
40. health state$ preference$.ti,ab.
41. Utility weight$.ti,ab.
42. (preference based measure$ or preference weight$ or utility preference$).ti,ab.
43. HSUV.ti,ab.
44. Rosser. ti,ab.
45. AQOL or assessment of quality of life.ti,ab.
46. economics/
47. Cost-Benefit analysis/
48. economics, hospital/
49. economics, medical/
50. economics, pharmaceutical/
51. economics, nursing/
52. (economic$ or pharmacoeconomic$).ti,ab.
53. (cost effectiveness or cost-effectiveness or cost utility or cost-Utility or cost benefit or cost-benefit or economic evaluation).ti,ab.
54. (CEA or CUA or CBA).ti,ab.
55. models, economic/
56. markov chains/
57. markov$.tw.
58. monte carlo method/
59. (monte adj carlo).tw.
60. decision tree/
61. decision analy$ .tw.
62. (decision adj2 (tree? or analys$)).tw.
63. or/7-62
64. Comment/
65. Letter/
66. Editorial/
67. Historical article.pt.
68. Animal/
69. Human/
70. 68 not (68 and 69)
71. or/64-67,70
72. 6 and 63

SYSTEMATIC REVIEW: RISK OF BIAS ASSESSMENT OF INDIVIDUAL HSPW ESTIMATES

There are no agreed methodological or reporting standards for HSPWs challenging objective critical appraisal. A novel, peer reviewed, critical appraisal checklist was therefore developed informed by NICE technical guidelines, theoretical considerations and recommendations from authorities in the field.([6-12](#_ENREF_6))

A methodological component approach, based on the Cochrane Collaboration’s tool for assessing risk of bias in randomised trials,([13](#_ENREF_13)) was taken evaluating the key steps of health state measurement and valuation of preferences separately. Selection and information bias were then assessed for each of these, along with an ‘other’ category to give 5 domains. Within each of these domains items potentially influencing risk of bias were specified, allowing a bias rating of low, high, unclear, or not applicable. As assessment of risk of bias requires judgement, items were not prescriptive e.g. the missingness mechanism is important rather than an arbitrary figure for missing data when determining the potential for selection bias.

Assessment focused on factors directly related to the risk of bias in that particular method of HSPW elicitation and did not judge the overall appropriateness of different techniques for obtaining preferences e.g the suitability of SG versus TTO, or different valuing populations. Depending on study design certain domains might not be applicable e.g. selection bias would not be relevant for health state description in studies using scenarios to measure HRQOL.

Relevance is a key factor in determining which HSPWs are appropriate for individual decision analysis models. By clearly reporting study characteristics review users will be able to judge which HSPW estimates are suitable for their setting. Relevance has therefore not been included as a specific component of bias assessment.

The risk of bias tool is detailed in Table 1.

**Table 1.** Risk of bias assessment instrument for HSPWs

|  | **Health state description & measurement** | | **Health state valuation** | | **Other** |
| --- | --- | --- | --- | --- | --- |
| **Domain:** | **Domain 1:** Selection bias (Representative population describing health sates?) | **Domain 2**: Information bias (Accurate and reproducible measurement of health states?) | **Domain 3:** Selection bias (Representative population valuing health sates?): | **Domain 4:** Information bias (Accurate and reproducible valuation of preference for health states?): | **Domain 5:** Other sources of bias: |
| **Items:** | •Response rates  •Loss to follow up  •Missing data | •Content validity  •Face validity  •Construct validity  •Responsiveness  •Reliability | •Response rates  •Loss to follow up  •Missing data | •Choice v feeling based valuation  •Credible extrapolation of health state valuations?  •Empirical validity of valuation method (against revealed, stated or hypothesised preferences) |  |
| **Criteria:** | *Low risk:* Health states measured in population representative of the target population. Random or census sample. High response rates, low loss to follow up, little missing data. Sensitivity analyses show results robust to selection bias. | *Low risk:* Well designed health state descriptions meeting consensus standards^[ref[^  Validated preference based multiattribute health description instrument used with adequate coverage for GOS states. | *Low risk:* Health states valued by a population representative of the target population. Random or census sample. High response rates, low loss to follow up, little missing data. Sensitivity analyses show results robust to selection bias. | *Low risk:* Direct valuation method (TTO,SG) performed appropriately.^[ref[^  Validated preference based multiattribute health description algorithm used.  Mapping function from non-preference based HRQOL instrument meets methodological standards.^[ref]^ | *Low risk:* No other sources of bias likely to influence results. |
|  | *High risk:* Health states measured in non-representative population secondary to:  •Non-random / convenience sample  •Low response rate  •High loss to follow up  •Missing data  Principled methods for handling missing data not used or sensitivity analyses indicate results not robust to missing data | *High risk:* Poorly designed health states not meeting methodological standards e.g. don’t include relevant health attributes.  Multiattribute health description instrument with inadequate coverage or used to measure health states from case notes. | *High risk:* Preferences obtained from non-representative population not compatible with target population, secondary to:  •Non-random / convenience sample  •Low response rate  •High loss to follow up  •Missing data  Principled methods for handling missing data not used or sensitivity analyses indicate results not robust to missing data | *High risk:* Direct valuation method (TTO,SG) performed inappropriately.^[ref[^  Un validated preference based multiattribute health description algorithm used.  Mapping function from non-preference based HRQOL instrument does not meet methodological standards.^[ref]^ | *High risk:* Other sources of bias expected to materially alter findings. |
|  | *Unclear:* Insufficient information reported to allow assessment | *Unclear:* Insufficient information reported to allow assessment | *Unclear:* Insufficient information reported to allow assessment | *Unclear:* Insufficient information reported to allow assessment | *Unclear:* Insufficient information reported to allow assessment |
|  | *Not applicable:* Health states measured using scenarios | *Not applicable:* Health states measured in patients | *Not applicable:* Health states measured using scenarios |  | *Not applicable:* No other appreciable sources of bias possible. |

MAPPING STUDY: adjusted limited dependent variable mixture models

The distribution of EQ5D preference values has a number of distinctive features challenging conventional approaches for statistical modelling. By definition, the maximum utility possible is perfect health and EQ5D values are consequently constrained to be less than one. The distribution is also limited at the lower end by a minimum possible value of -0.594 for the worst possible health state (33333, representing extreme problems in all 5 EQ5D health dimensions). The probability density function between these values is discontinuous, multimodal and skewed. In most disease areas there is usually a large probability density at full health. The next possible EQ5D value is 0.883 (the 11211 health state, corresponding to some problems with usual activities) resulting in a large gap at the upper end of the distribution. Although other smaller gaps exist across the possible range of EQ5D values, particularly at 0.45 as a result of the N3 term, the remainder of the distribution is usually considered to be continuous. There are usually at least two modes located around EQ5D values of 0.2 and 0.7. However, the exact number of modes, and their associated skewness and kurtosis, will vary according to the patient characteristics and disease area being assessed. These distributional characteristics normally remain after conditioning on patient or disease characteristics.([6](#_ENREF_6), [14](#_ENREF_14), [15](#_ENREF_15))

A number of statistical approaches have been employed in mapping studies estimating EQ5D HSPWs from clinical outcome scores. These have included linear regression, generalised linear modelling, tobit regression, symmetrically trimmed least squares regression, censored least absolute deviation (CLAD) models, adjusted limited dependent variable models, response mapping, and 2 part logistic – linear regression models.([16](#_ENREF_16), [17](#_ENREF_17)) Each of these techniques have been shown to be sub-optimal with problems including prediction of impossible values beyond the EQ5D range, under-prediction of high EQ5D values, and systematic over-prediction at the lower end of the EQ5D scale. In response to these concerns a novel modelling approach based on semi-parametric mixture models, the adjusted limited dependent variable mixture model, has recently been developed and demonstrated to have superior performance to previously used techniques.([14](#_ENREF_14))

Mixture models can be used for 2 main purposes: identifying and describing heterogeneous groups in a population; or to provide a semi-parametric framework to model challenging distributions as in the current study.([18](#_ENREF_18), [19](#_ENREF_19)) Mixture models combine a number of normal distributions to produce extremely flexibly shaped distributions which can incorporate multi-modality, and extreme kurtosis or skewness.([18](#_ENREF_18), [19](#_ENREF_19)) Restricting these models, with a limited dependent variable covering the range of possible EQ5D values, adjusting for the large components of one’s, and rounding of predicted values above 0.883 to account for the gap between full health and the next possible score, allows all important features of the EQ5D distribution to be accounted for. The population is considered to be composed of several distinct groups (often called latent classes, mixtures, classes, or components) which have different distributions of EQ5D values. Mixture models determine the probability of latent class membership using a multinomial logit model and then estimate a normal distribution for each group. The relationship between patient characteristics and the probability of latent class membership, and the form of each normal distribution, can be incorporated into the model. The mean EQ5D is then calculated based on an average of the predictions from the normal distribution for each latent class, weighted by the probabilities of component membership.([14](#_ENREF_14))

MAPPING STUDY: Modelling strategy

The modelling strategy followed recommendations for mixture modelling and the development of clinical prediction models.([14](#_ENREF_14), [15](#_ENREF_15), [20](#_ENREF_20)) As recommended by Hernandez (2014) simple models were initially evaluated with the number of covariates and components progressively increased.([21](#_ENREF_21))

Two groups of models were developed. Previous TBI economic models have overwhelmingly used cohort methodology, examining mixed male/female populations of a specified nominal age.([22-27](#_ENREF_22)) Firstly, an initial model was therefore developed with 12 month EQ5D as the dependent variable and GOS category and age as fixed explanatory variables. Patient level simulations or trial based economic evaluations may also require HSPWs for GOS categories conditional on other patient characteristics. Secondly, age, gender, co-morbidities, and the presence of extra-cranial injury were considered to represent important patient variables likely to be important when characterising TBI populations, and were therefore evaluated as further covariates in an additional more detailed model. Previous economic models have exclusively used basic GOS for defining health states and interest therefore focused on the 5 level GOS version.([28](#_ENREF_28)) Models for the extended GOS were developed in additional analyses.

Inclusion of explanatory variables was primarily determined by clinical considerations and all specified covariates were judged to be important in influencing utility after TBI. Omission of clearly non-significant variables (p>0.5) predicting utility in particular components, or the probability of membership of specific components, was decided on an individual basis and guided by measures of model goodness of fit. Polynomial terms were evaluated to account for further non-linearity in the relationship between age and mean EQ5D. Coding of covariates is summarised in Table 2. Secondary models examining extended GOS categories as dummy variables did not converge, necessitating the use of a continuous term to predict latent class membership. A quadratic term was evaluated to assess non-linearity but was non-significant.

The appropriate number of latent classes was determined by considering the proportion of cases in each component, changes in information criterion statistics, and whether all important parts of the EQ5D distribution were modelled. Higher Bayesian Information Criteria, emergence of very small components likely to include only outlying data, and coverage of all important regions of the EQ5D distribution argued against increased complexity.

**Table 2. Coding of covariates in adjusted limited dependent variable mixture models**

| Explanatory variable | Coding | Rationale |
| --- | --- | --- |
| Age | Continuous linear term | Categorisation would lose information.  Polynomial terms non-significant |
| Co-morbidity | Categorical, based on ASA scoring:  • No/ mildly limiting  • Limiting /critical | Clinically meaningful categories within constraints of data |
| Extra-cranial injury | Categorical, based on AIS:  • No non-head injury AIS≥3  • Non-head injury AIS≥3 | Clinically meaningful categories |
| Gender | Male/female | Natural categorisation |
| Basic GOS | Categorical:  • Vegetative state  • Severe disability  • Moderate disability  • Good recovery | Death has a utility of zero and therefore does not need to be predicted. Defined categorisation of outcome measure. |
| Extended GOS | Categorical to predict mean EQ5D within latent class  Continuous linear term to predict latent class membership | Defined categorisation of outcome measure. Non-convergence of model if entered as categorical variable to predict latent class membership. Polynomial terms non-significant |

ASA: American Society of Anaesthesiologists; AIS: Abbreviated Injury Score

The parameters of mixture models are estimated using maximum likelihood with an expectation maximisation algorithm. From an initial set of speculative starting values a probability distribution for possible component memberships (or ‘completions’) is computed using the current parameters. New parameters are then determined based on the current completions, with the estimated model slightly improving. After a number of iterations the algorithm converges to maximise the expected log-likelihood of the data.([29](#_ENREF_29)) A well known problem is that the maximum likelihood estimation is very sensitive to the initial values and can become trapped at local maxima in the likelihood function, meaning that the model’s estimated parameters are not correct. To ensure a consistent solution was achieved, with the maximum possible likelihood function, a number of steps were taken: Initial starting values were varied, a constant only model was fitted and these starting values used to develop subsequent models, and simulated annealing was implemented to identify the most appropriate staring values.([15](#_ENREF_15), [21](#_ENREF_21), [30](#_ENREF_30))

Goodness of model fit was evaluated using the Bayesian and Akaike’s information criterion statistics, mean absolute error, root mean squared error, and visual comparison of predicted and observed values across the range of GOS categories. Adjusted limited dependent variable mixture modelling was implemented using the *aldmm* module in Stata 12.1, using the *siman* module for simulated annealing.([21](#_ENREF_21))

MAPPING STUDY: secondary analyses

A number of secondary analyses were additionally performed:

- Missing data was investigated by examining case-wise and variable-wise missingness and comparing patient characteristics between included and excluded cases.
- General population preference weights for EQ5D health state have been derived for a number of different countries. Mean EQ5D values for basic GOS categories were therefore also calculated based on a range of international tariffs to increase the generalisability of the results.
- Primary analyses were repeated for the extended GOS.

**Supplementary results**

SYSTEMATIC REVIEW: CHARACTERISTICS OF INCLUDED STUDIES

**Table 3. Characteristics of included studies**

| **Study** | **Design** | **Health states examined** | **Population in which health state is measured/described** | **N for health state measurement** | **Method used to describe/measure health state** | **Population providing preferences for health state** | **N of health state valuation** | **Method used to determine preferences for HRQOL of health state** |
| --- | --- | --- | --- | --- | --- | --- | --- | --- |
| Kosty 2013([31](#_ENREF_31)) | Direct determination of preferences using hypothetical clinical scenarios | Extended GOS categories. | Scenarios labelled as head injuries. | N/A | Descriptive scenarios of extended GOS categories | US general public | n=101  50% college students,  54% male, Mean age 40.4 years | Direct valuation using standard gamble |
| Smits 2010([24](#_ENREF_24), [32](#_ENREF_32)) | Indirect determination of preferences for health states measured from patients using generic multi-attribute preference based health description instrument | Basic GOS categories | Adults >16 years with mild blunt complicated TBI (GCS 13-15).  Presenting to Dutch university hospitals 2002-2004. | n=87 | EQ5D | Dutch general population | n=309 | Indirect valuation using valuation algorithm (derived using TTO for limited number of described health states) applied to measured health states. |
| Dijkers 2004([33](#_ENREF_33)) | Indirect determination of preferences for health states measured from scenarios using generic multi-attribute preference based health description instrument | ‘Mild’, ‘moderate’ and ‘severe’ TBI corresponding to GOS categories 5,4,3. | Scenarios labelled as head injuries. | N/A | Descriptive scenarios scored by author against QWB, HUI3 | Australian general public (QWB)  Canadian general public (HUI) | n=866, n=504 | Indirect valuation using valuation algorithm (derived from US/Canadian general population using MAUT/VAS) applied to measured health states. |
| Tsauo 1999([34](#_ENREF_34)) | Indirect determination of preferences for health states measured from patients using generic multi-attribute preference based health description instrument. | Basic GOS categories | Adult and paediatric patients with head injury arising from motorcycle accidents | n=99 | Rosser health classification instrument | UK general population and patients | n=70 | Indirect valuation using valuation matrix (derived using magnitude estimation from limited number of described health states) |
| Aoki 1998([35](#_ENREF_35)) | Direct determination of preferences using hypothetical clinical scenarios | Basic GOS categories 1-5, with additional 6^th^ category added for ‘perfect recovery’ | Scenarios unlabelled. HSPWs derived for non-TBI study. | N/A | Descriptive scenarios of GOS categories | Japanese health professionals | n=140  59% medical students, 16% doctors, 21% nurses,4% technicians | Direct valuation using standard gamble |

### Detailed risk of bias assessments for included GOS HSPW studies

| **Kosty 2012(**[**31**](#_ENREF_31)**)** | | | | | |
| --- | --- | --- | --- | --- | --- |
|  | **Health state description & measurement** | | **Health state valuation** | | **Other** |
| **Risk of bias domain:** | **Domain 1:** Selection bias (Representative population describing health sates?) | **Domain 2**: Information bias (Accurate and reproducible measurement of health states?) | **Domain 3:** Selection bias (Representative population valuing health sates?): | **Domain 4:** Information bias (Accurate and reproducible valuation of preference for health states?): | **Domain 5:** Other sources of bias: |
| **Bias judgement:** | Not applicable | Low risk | High risk | Low risk | Low risk |
| **Support for judgement:** | Health states measured using scenarios | Well designed health state descriptions meeting consensus standards.  GOS category for Good Recovery Upper assigned perfect health.  Relevant functional dimensions of GOS categories included.  Heterogeneity of GOS states not fully accounted for e.g. Moderate disability addresses limitations in work only. | Target population providing preferences was the USA general population.  The study sample comprised a non-representative sample of the general population.  50% of sample was young adults attending college. | Direct valuation method using standard gamble performed appropriately.  Structured interviews use to obtain preferences. Full details on methods not described e.g. use of props or training exercises.  GOS category for Good Recovery Upper assigned perfect health. | No other sources of bias identified likely to influence results. |

| **Smits 2010(**[**24**](#_ENREF_24)**,** [**32**](#_ENREF_32)**)** | | | | | |
| --- | --- | --- | --- | --- | --- |
|  | **Health state description & measurement** | | **Health state valuation** | | **Other** |
| **Domain:** | **Domain 1:** Selection bias (Representative population describing health sates?) | **Domain 2**: Information bias (Accurate and reproducible measurement of health states?) | **Domain 3:** Selection bias (Representative population valuing health sates?): | **Domain 4:** Information bias (Accurate and reproducible valuation of preference for health states?): | **Domain 5:** Other sources of bias: |
| **Bias judgement:** | High risk | Low risk | Low risk | Low risk | Low risk |
| **Support for judgement:** | Target population for measuring health states was a sample of consecutive patients with complicated mild TBI.  Substantial loss to follow up for measurement using EQ5D (58%).  Differences in case mix between included and excluded patients in terms of gender, age and TBI pathology. | Validated preference based multiattribute health description instrument used with adequate coverage for GOS states – EQ5D.  Telephone interviews used to measure health states. Full details on methods not described e.g. structured interviews. | Health states valued by a sample of Dutch population representative of the target Dutch general population. | Preferences obtained indirectly using Dutch EQ5D tariff; derived from study meeting methodological standards for obtaining Preferences obtained indirectly using Dutch EQ5D tariff an algorithm for preferences. | No other sources of bias identified likely to influence results. |

| **Djikers 2004(**[**33**](#_ENREF_33)**)** | | | | | |
| --- | --- | --- | --- | --- | --- |
|  | **Health state description & measurement** | | **Health state valuation** | | **Other** |
| **Risk of bias domain:** | **Domain 1:** Selection bias (Representative population describing health sates?) | **Domain 2**: Information bias (Accurate and reproducible measurement of health states?) | **Domain 3:** Selection bias (Representative population valuing health sates?): | **Domain 4:** Information bias (Accurate and reproducible valuation of preference for health states?): | **Domain 5:** Other sources of bias: |
| **Bias judgement:** | Not applicable | High risk | High risk | Low risk | Low risk |
| **Support for judgement:** | Health states measured using scenarios | Un-validated health states based on expert opinion.  Relevant functional dimensions of GOS categories included. | Target population providing preferences is health professionals.  The study sample comprised the study author only. | Preferences obtained indirectly using HUI-3 and QWB scoring algorithms. | No other sources of bias identified likely to influence results. |

| **Tsauo 1999(**[**34**](#_ENREF_34)**)** | | | | | |
| --- | --- | --- | --- | --- | --- |
|  | **Health state description & measurement** | | **Health state valuation** | | **Other** |
| **Risk of bias domain:** | **Domain 1:** Selection bias (Representative population describing health sates?) | **Domain 2**: Information bias (Accurate and reproducible measurement of health states?) | **Domain 3:** Selection bias (Representative population valuing health sates?): | **Domain 4:** Information bias (Accurate and reproducible valuation of preference for health states?): | **Domain 5:** Other sources of bias: |
| **Bias judgement:** | High risk | High risk | Unclear | Unclear | Low risk |
| **Support for judgement:** | Target population for measuring health states was patients with head injury following motorcycle accidents.  Study sample suffered from substantial loss to follow up (75%) with risk of selection bias if unrepresentative patients described health states | Patients asked to describe health state several years previously with concomitant risk of recall bias.  Un-validated translation of preference based multi-attribute health description instrument used with adequate coverage for GOS states (Rosser Index of health related quality of life).  Telephone interviews used to measure health states. Full details on methods not described e.g. structured interviews. | Population valuing health states in the Rosser Index of health related quality of life not reported. | Direct valuation method using standard gamble.  Details of valuation exercises not published. | No other sources of bias identified likely to influence results. |

| **Aoki 1998(**[**35**](#_ENREF_35)**)** | | | | | |
| --- | --- | --- | --- | --- | --- |
|  | **Health state description & measurement** | | **Health state valuation** | | **Other** |
| **Domain:** | **Domain 1:** Selection bias (Representative population describing health sates?) | **Domain 2**: Information bias (Accurate and reproducible measurement of health states?) | **Domain 3:** Selection bias (Representative population valuing health sates?): | **Domain 4:** Information bias (Accurate and reproducible valuation of preference for health states?): | **Domain 5:** Other sources of bias: |
| **Bias judgement:** | Not applicable | High risk | Unclear | Low risk | Low risk |
| **Support for judgement:** | Health states measured using scenarios | Health state descriptions do not meet consensus methodological standards.  Brief health state descriptions which don’t include all relevant functional dimensions of GOS categories.  Heterogeneity of GOS states not accounted for e.g. Moderate disability doesn’t address limitations in leisure activities | Target population is Japanese health professionals.  Insufficient information reported on inclusion criteria and sample selection to allow assessment. | Direct valuation method using standard gamble performed appropriately.  Structured interviews use to obtain preferences. Full details on methods not described e.g. use of props or training exercises. | No other sources of bias identified likely to influence results. |

mapping study: derivation of study sample

Between July 2008 and June 2013 13,742 patients with major trauma were identified as eligible for inclusion in VSTR of which 68 refused permission for inclusion of data. From this source population 6,236 patients had significant TBI. Of these 1,282 cases had incomplete information on 12 month EQ5D or 12 month GOS and were excluded from complete case analyses. A further 1,517 patients had died by 12 months and were therefore not considered in the initial predictive models’ derivation sample. The origin of the final study sample of 3,437 is presented schematically in Figure 1.


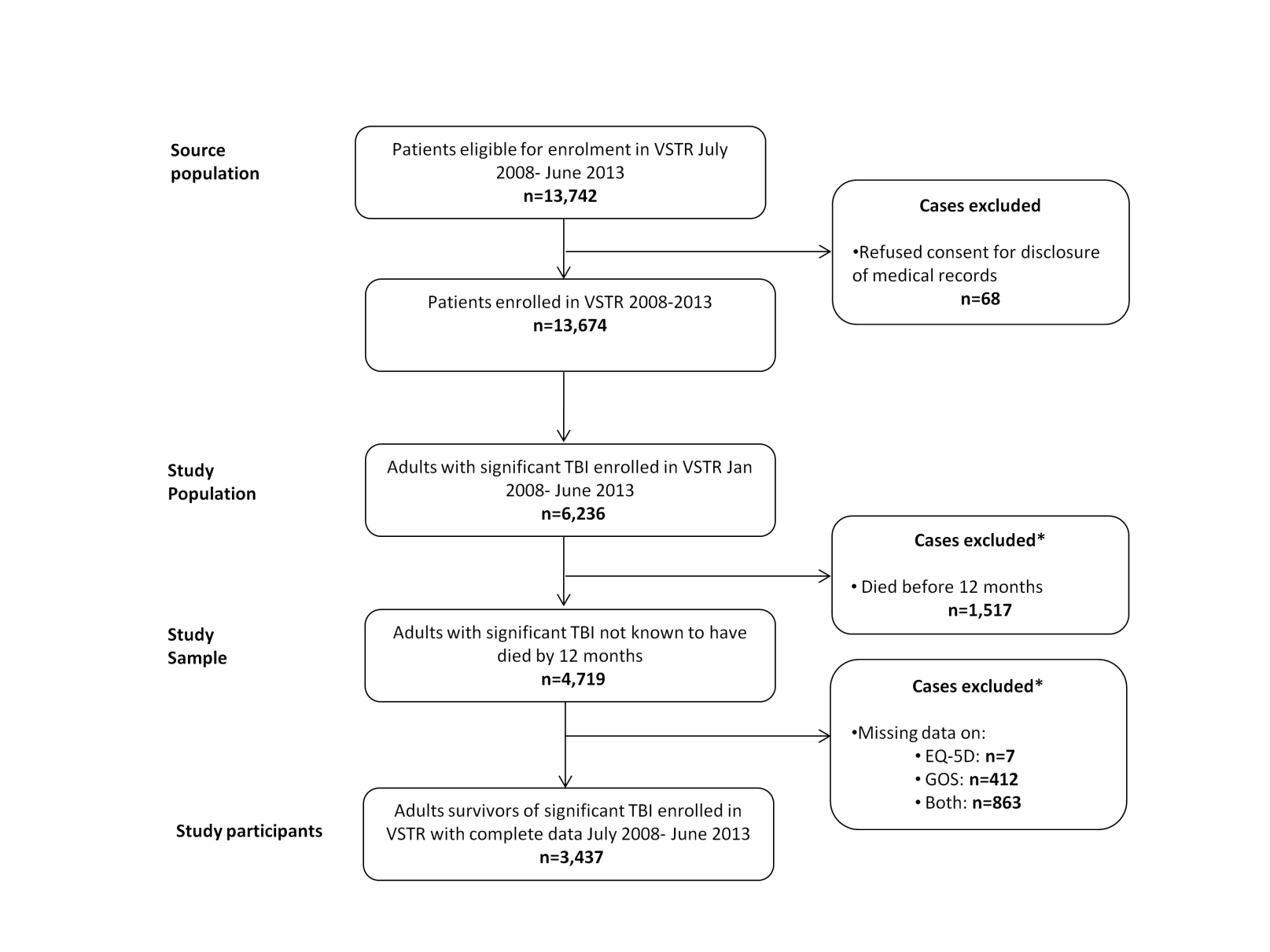


**Figure 1. Flow chart showing derivation of study sample used to develop the intital predictive model including basic GOS category and age.**

mapping study: patient characteristics

The median age of the study sample was 50 years (Inter-quartile range 29-72) with males accounting for 71.3% of cases (95% CI 69.8-72.9%) The majority of patients were healthy prior to their TBI (72.9%), but a notable minority had a preceding limiting or critical systemic illness (27.1%). The most common mechanisms of injury were falls (46.8%), transport related accidents (41.1%), and assaults (11.3%). Clinical presentation as mild TBI preponderated with the median admission GCS recorded as 14 (inter-quartile range 13-15). However, most patients were ultimately diagnosed with an AIS head injury severity score of 4 (40.4%), or 5 (22.5%) injuries. A substantial minority of patients presented with a concomitant extra-cranial injury (30.8%, 95% CI 29.3-32.4%). The median ISS was 21 (inter-quartile range 16-26). Patient characteristics are summarised in Table 4.

**Table 4. Comparison of patient characteristics stratified by basic GOS category**

| Patient characteristic | Overall | Stratified by basic GOS category | | | | p-value* |
| --- | --- | --- | --- | --- | --- | --- |
|  |  | **GOS 2:**  **PVS** | **GOS 3:**  **Severe disability** | **GOS 4:**  **Moderate disability** | **GOS 5:**  **Good recovery** |  |
| Number of patients | 3,437 | 6 | 900 | 1,222 | 1,309 |  |
| Age at injury  (Years, median, IQR) | 50  (29-72) | 52.5  (47-69) | 73.5  (48-83) | 39  (25-56 | 48  (56-68) | <0.001 |
| Male gender  (%, 95% CI) | 71.3  (69.8-72.9) | 83.3  (50.7-1.0) | 58.0  (54.8-61.2) | 77.4  (75.1-79.8) | 74.8  (72.4-77.1) | <0.001 |
| **Co-morbidities**  (%, 95% CI): |  |  |  |  |  |  |
| • Limiting or critical systemic illness | 27.1  (25.5-28.7) | 60.0  (12.0-1.0) | 49.3  (45.8-52.8) | 16.3  (14.1-18.4) | 22.0  (19.7-24.4) | <0.001 |
| **Head region AIS** (%, 95% CI): |  |  |  |  |  |  |
| • AIS 3 | 37.0  (35.4-38.7) | - | 24.1  (40.8-46.4) | 43.6  (40.8-46.4) | 40.0  (37.3-42.6) | <0.001 |
| • AIS 4 | 40.4  (38.7-42.0) | 50.0  (6.1-93.8) | 45.2  (42.0-48.5) | 37.3  (34.6-40.0) | 39.9  (37.2-42.5) |  |
| • AIS 5 | 22.5  (21.1-23.9) | 50.0  (6.1-93.8) | 30.6  (27.5-33.6) | 19.0  (16.8-21.2) | 20.2  (18.0-22.3) |  |
| • AIS 6 | ~~-~~ | ~~-~~ | ~~-~~ | ~~-~~ | ~~-~~ |  |
| • AIS 9† | 0.05  (0.0-0.01) | ~~-~~ | 0.11  (0.0-0.3) | 0.08  (0.0-0.2) | - |  |
| Admission GCS  (median, IQR) | 14  (13-15) | 8  (3-10) | 14  (12-15) | 14  (11-15) | 15  (14-15) | <0.001 |
| Extra-cranial injury  (n=3,436, AIS≥3, %, 95% CI) | 30.8  (29.3-32.4) | 33.3  (0.0-74.7) | 27.3  (24.3-30.2) | 41.8  (39.0-44.6) | 23.0  (20.7-25.3) | <0.001 |
| ISS  (median, IQR) | 21  (16-26) | 29  (24-35) | 21  (17-26) | 22  (17-27) | 17  (16-25) | <0.001 |
| 12 month EQ5D  (mean, 95% CI) | 0.68  (0.67-0.69) | -0.178  (-0.33- -0.25) | 0.382  (0.36-0.41) | 0.674  (0.66-0.69) | 0.894  (0.89-0.90) | <0.001 |

### *p-value represents the probability of obtaining results at least as extreme as those observed, assuming the null hypothesis of no difference in patient characteristic across GOS categories is true. † AIS 9 refers to death ascribed to head injury without further substantiation of injuries.

GOS: Glasgow Outcome Scale, AIS: Abbreviated Injury Scale, ISS: Injury Severity Score, GCS: Glasgow Coma Scale, IQR: Inter-quartile range, 95% CI: 95% confidence interval, EQ5D: European Quality of Life-5 Dimensions

MAPPING STUDY: COEFFICENTS FOR PRIMARY PREDICTIVE MODELS

**Table 5. Coefficients for the initial adjusted limited dependent variable mixture model predicting 12 month EQ5D HSPWs from basic GOS category and age.**

| Variable* | Coefficient | SE | p-Value | 95% Confidence Interval | |
| --- | --- | --- | --- | --- | --- |
| **Explanatory variables within component 1** | | | | | |
| Vegetative state | -0.524 | 0.077 | 0.000 | -0.675 | -0.373 |
| Severe disability | -0.196 | 0.049 | 0.000 | -0.293 | -0.099 |
| Moderate disability | -0.053 | 0.051 | 0.296 | -0.154 | 0.047 |
| Age | 0.001 | 0.005 | 0.815 | -0.008 | 0.010 |
| Constant | 0.280 | 0.053 | 0.000 | 0.176 | 0.385 |
| **Explanatory variables within component 2** | | | | | |
| Vegetative state | -0.778 | 0.114 | 0.000 | -1.002 | -0.554 |
| Severe disability | -0.001 | 0.092 | 0.991 | -0.182 | 0.180 |
| Moderate disability | 0.041 | 0.077 | 0.598 | -0.111 | 0.192 |
| Age | -0.011 | 0.002 | 0.000 | -0.015 | -0.008 |
| Constant | 0.844 | 0.054 | 0.000 | 0.737 | 0.950 |
| **Explanatory variables within component 3** | | | | | |
| Vegetative state† | - | - | - | - | - |
| Severe disability | -0.337 | 0.047 | 0.000 | -0.429 | -0.245 |
| Moderate disability | -0.282 | 0.036 | 0.000 | -0.353 | -0.211 |
| Age | -0.012 | 0.002 | 0.000 | -0.015 | -0.009 |
| Constant | 1.002 | 0.020 | 0.000 | 0.962 | 1.042 |
| **Explanatory variables explaining the probability of component 1 membership** | | | | | |
| Vegetative state | 20.562 | 1.441 | 0.000 | 17.738 | 23.386 |
| Severe disability | 3.962 | 0.298 | 0.000 | 3.378 | 4.546 |
| Moderate disability | 2.785 | 0.333 | 0.000 | 2.133 | 3.437 |
| Age | 0.002 | 0.039 | 0.957 | -0.075 | 0.079 |
| Constant | -3.143 | 0.273 | 0.000 | -3.679 | -2.607 |
| **Explanatory variables explaining the probability of component 2 membership** | | | | | |
| Vegetative state | 17.000 | - | - | - | - |
| Severe disability | 0.697 | 0.244 | 0.004 | 0.219 | 1.174 |
| Moderate disability | 1.285 | 0.234 | 0.000 | 0.827 | 1.744 |
| Age | 0.077 | 0.033 | 0.021 | 0.012 | 0.142 |
| Constant | -1.029 | 0.736 | 0.162 | -2.471 | 0.413 |
| **Sigma** |  |  |  |  |  |
| Sigma 1 | 0.212 | 0.009 |  | 0.195 | 0.230 |
| Sigma 2 | 0.086 | 0.019 |  | 0.056 | 0.131 |
| Sigma 3 | 0.061 | 0.013 |  | 0.041 | 0.092 |

*Basic GOS coded as indicator variable with GOS 5, good recovery, as the baseline category. Basic GOS category 1, death, not modelled as this will equal zero by definition. †There was a zero probability of membership of class 3 if in persistent vegetative state. This coefficient was therefore constrained to zero.

Covariance matrix available on request. A stata do file allowing calculation of mean EQ-5D with 95% confidence intervals for a given basic GOS and age category is supplied as an additional file.

**Table 6. Model coefficients for detailed model including basic GOS**

| Variable* | Coefficient | SE | p-Value | 95% Confidence Interval | |
| --- | --- | --- | --- | --- | --- |
| **Explanatory variables within component 1** | | | | | |
| Age | -0.001 | 0.005 | 0.819 | -0.011 | 0.009 |
| Female gender | -0.030 | 0.019 | 0.107 | -0.067 | 0.007 |
| Limiting or critical co-morbidity | -0.004 | 0.020 | 0.860 | -0.044 | 0.036 |
| Major extra-cranial injury | -0.056 | 0.020 | 0.006 | -0.096 | -0.016 |
| Vegetative state | -0.548 | 0.074 | 0.000 | -0.692 | -0.403 |
| Severe disability | -0.160 | 0.047 | 0.001 | -0.251 | -0.069 |
| Moderate disability | -0.035 | 0.047 | 0.455 | -0.128 | 0.057 |
| Constant | 0.291 | 0.052 | 0.000 | 0.190 | 0.392 |
| **Explanatory variables within component 2** | | | | | |
| Age | -0.010 | 0.002 | 0.000 | -0.013 | -0.007 |
| Female gender | -0.032 | 0.008 | 0.000 | -0.048 | -0.017 |
| Limiting or critical co-morbidity | -0.023 | 0.009 | 0.011 | -0.040 | -0.005 |
| Major extra-cranial injury | -0.028 | 0.009 | 0.002 | -0.046 | -0.010 |
| Vegetative state | -0.871 | 0.075 | 0.000 | -1.018 | -0.724 |
| Severe disability | -0.039 | 0.042 | 0.356 | -0.121 | 0.043 |
| Moderate disability | 0.000 | 0.032 | 0.990 | -0.062 | 0.063 |
| Constant |  |  |  |  |  |
| **Explanatory variables within component 3** | | | | | |
| Age | -0.014 | 0.002 | 0.000 | -0.018 | -0.009 |
| Female gender | -0.009 | 0.008 | 0.246 | -0.025 | 0.007 |
| Limiting or critical co-morbidity | -0.018 | 0.009 | 0.049 | -0.035 | 0.000 |
| Major extra-cranial injury | -0.029 | 0.011 | 0.007 | -0.051 | -0.008 |
| Vegetative state | 0.000 |  |  |  |  |
| Severe disability | -0.348 | 0.024 | 0.000 | -0.394 | -0.302 |
| Moderate disability | -0.300 | 0.020 | 0.000 | -0.338 | -0.262 |
| Constant |  |  |  |  |  |
| **Explanatory variables explaining the probability of component 1 membership** | | | | | |
| Age | -0.035 | 0.037 | 0.343 | -0.106 | 0.037 |
| Limiting or critical co-morbidity | 0.721 | 0.229 | 0.002 | 0.272 | 1.170 |
| Vegetative state | 20.442 | 1.442 | 0.000 | 17.616 | 23.269 |
| Severe disability | 3.938 | 0.297 | 0.000 | 3.355 | 4.520 |
| Moderate disability | 3.063 | 0.308 | 0.000 | 2.459 | 3.666 |
| Constant | -3.011 | 0.275 | 0.000 | -3.550 | -2.472 |
| **Explanatory variables explaining the probability of component 2 membership** | | | | | |
| Age | 0.055 | 0.033 | 0.093 | -0.009 | 0.119 |
| Limiting or critical co-morbidity | 0.566 | 0.211 | 0.007 | 0.153 | 0.978 |
| Vegetative state | 17.000 | - | - | - | - |
| Severe disability | 0.403 | 0.320 | 0.209 | -0.225 | 1.031 |
| Moderate disability | 1.291 | 0.253 | 0.000 | 0.794 | 1.787 |
| Constant | 0.055 | 0.033 | 0.093 | -0.009 | 0.119 |
| **Sigma** |  |  |  |  |  |
| Sigma 1 | 0.203 | 0.009 | - | 0.186 | 0.222 |
| Sigma 2 | 0.094 | 0.007 | - | 0.081 | 0.110 |
| Sigma 3 | 0.056 | 0.008 | - | 0.042 | 0.075 |

*Basic GOS coded as indicator variable with GOS 5, good recovery, as the baseline category. Basic GOS category 1, death, not modelled as this will equal zero by definition. Covariance matrix available from author on request. A stata do file allowing calculation of mean EQ-5D with 95% confidence intervals for a given basic GOS and covariates is supplied as an additional file.

MAPPING STUDY: RESULTS OF SECONDARY ANALYSES

*Characteristics of excluded patients*

There was a moderate proportion of missing data for important study variables, ranging from 0% for age and sex to 27.0% for 12 month EQ5D (Table 7). Case-wise missingness for the initial predictive model ranged from 72.8% of cases with no missing data to 18.3% of patients missing data on both 12 month EQ5D and basic GOS. Case-wise missingness for the detailed predictive model varied from 65.5% of cases having no missing data on any variable to 1.9% of cases missing data on 3 out of the 6 included variables (Table 8).

**Table 7. Missing data levels for variables included in models predicting 12 month EQ5D**

| **Patient characteristic**  **(Total sample size 4,719)** | **Data incompleteness***  **Cases with missing data, (%)** |
| --- | --- |
| 12 month EQ5D | 1,275 (27.0%) |
| 12 month GOS | 870 (18.4%) |
| Age | 0 (0%) |
| Gender | 0 (0%) |
| Co-morbidity | 480 (10.2%) |
| Extra-cranial injury | 2 (0.04%) |

**Table 8. Missing data levels for variables included in models predicting 12 month EQ5D**

| **Variables missing from initial model** | **Number of cases, (%)** | **Variables missing from detailed model** | **Number of cases, (%)** |
| --- | --- | --- | --- |
| None | 3,437 (72.8%) | None | 3,089 (65.5%) |
| GOS | 7 (0.2%) | EQ5D | 370 (7.8%) |
| EQ5D | 412 (8.7%) | Co-morbidity | 347 (7.4%) |
| EQ5D and GOS | 863 (18.3%) | EQ5D and GOS | 773 (16.4%) |
|  |  | Co-morbidity and extra-cranial | 1 (0.02%) |
|  |  | Co-morbidity and extra-cranial injury | 1 (0.02%) |
|  |  | EQ5D and co-morbidity | 42 (0.9%) |
|  |  | EQ5D, GOS, co-morbidity | 89 (1.9%) |
|  |  | EQ5D, GOS, extra-cranial injury | 1 (0.02%) |

Patients excluded from the available case analyses due to missing data had broadly similar characteristics to included patients, as detailed in Table 9. The only clinically and statistically significant differences were that excluded patients were marginally younger with slightly less severe injuries.

**Table 9. Characteristics of included and excluded cases**

|  | Initial model | |  |  | Detailed model | |  |
| --- | --- | --- | --- | --- | --- | --- | --- |
| **Patient characteristic** | **Included** | **Excluded** | **p-value** |  | **Included** | **Excluded** | **p-value** |
| Number of patients | 3,437 | 1,282 |  |  | 3,089 | 1,630 |  |
| Age at injury  (Years, median, IQR) | 50  (29-72) | 45  (27-67) | 0.001 |  | 50  (29-72) | 46  (28-67) | <0.001 |
| Male gender  (%, 95% CI) | 71.3  (69.8-72.9) | 73.0  (70.6-75.4) | 0.26 |  | 70.8  (69.2-72.4) | 73.6  (71.5-75.8) | 0.04 |
| **Co-morbidities**  (%, 95% CI): |  |  |  |  |  |  |  |
| • Limiting or critical systemic illness | 27.1  (25.5-28.7) | 28.1  (25.5-30.7)  n=1,150 | 0.52 |  | 27.1  (25.5-28.7) | 28.1  (25.5-30.7) | 0.52 |
| **Head region AIS** (%, 95% CI): |  |  |  |  |  |  |  |
| • AIS 3 | 37.0  (35.4-38.7) | 26.2  (33.6-38.8) | 0.53 |  | 36.9  (35.2-38.6) | 33.7  (34.3-39.0) | 0.37 |
| • AIS 4 | 40.4  (38.7-42.0) | 42.0  (39.3-44.7) |  |  | 40.6  (38.9-42.4) | 41.2  (38.8-43.6) |  |
| • AIS 5 | 22.5  (21.1-23.9) | 21.6  (19.4-23.9) |  |  | 22.5  (21.0-23.9) | 21.9  (19.9-23.9) |  |
| • AIS 6 | ~~-~~ | ~~-~~ |  |  | - | - |  |
| • AIS 9† | 0.05  (0.0-0.01) | 0.16  (0.0-0.37) |  |  | 0.03  (0.0-0.09) | 0.19  (0.0-0.39) |  |
| Admission GCS  (median, IQR) | 14  (13-15) | 14  (13-15)  n=1,237 | 0.72 |  | 14  (13-15) | 14  (13-15)  n=1,566 | 0.002 |
| Extra-cranial injury  ( AIS≥3, %, 95% CI) | 30.8  (29.3-32.4)  n=3,436 | 25.5  (23.1-27.9)  n=1,281 | <0.001 |  | 30.6  (28.9-32.2) | 27.1  (25.0-29.3)  n=1,628 | 0.02 |
| ISS  (median, IQR) | 21  (16-26) | 19  (16-26)  n=1,281 | 0.001 |  | 20  (16-26) | 20  (16-26)  n=1,628 | 0.05 |
| 12 month EQ5D  (mean, 95% CI) | 0.68  (0.67-0.69) | 0.71  (0.30-1.0)  n=7 | 0.51 |  | 0.68  (0.67-0.69) | 0.68  (0.65-0.72) | 0.89 |

*Non-UK tariff EQ-5D estimates for GOS categories*

**Table 10. Mean EQ5D values for basic GOS categories using a range of international preference tariffs**

|  | Glasgow Outcome Scale category: Mean (sd) | | | | |
| --- | --- | --- | --- | --- | --- |
|  | **GOS 1:**  **Death** | **GOS 2:**  **Vegetative state** | **GOS 3:**  **Severe disability** | **GOS 4:**  **Moderate disability** | **GOS 5:**  **Good recovery** |
| **n=** | - | 6 | 900 | 1,222 | 1,309 |
| **United Kingdom** | 0  (-) | -0.178  (0.19) | 0.382  (0.35) | 0.675  (0.27) | 0.894  (0.16) |
| **Denmark** | 0  (-) | -0.478  (0.20) | 0.489  (0.29) | 0.711  (0.20) | 0.900  (0.14) |
| **Germany** | 0  (-) | 0.327  (0.14) | 0.555  (0.30) | 0.811  (0.22) | 0.946  (0.11) |
| **Netherlands** | 0  (-) | 0.125  (0.18) | 0.471  (0.29) | 0.696  (0.25) | 0.905  (0.15) |
| **Spain** | 0  (-) | -0.397  (0.13) | 0.350  (0.39) | 0.718  (0.25) | 0.917  (0.14) |
| **Japan** | 0  (-) | 0.072  (0.10) | 0.529  (0.20) | 0.710  (0.14) | 0.884  (0.14) |
| **United States** | 0  (-) | 0.092  (0.96) | 0.540  (0.24) | 0.754  (0.17) | 0.914  (0.12) |
| **Zimbabwe** | 0  (-) | 0.184  (0.15) | 0.529  (0.20) | 0.710  (0.14) | 0.884  (0.14) |
| **p-values** | - | <0.001 | <0.001 | <0.001 | <0.001 |

**Table 11. Mean EQ5D values for extended GOS categories using a range of international preference tariffs**

|  | Glasgow Outcome Scale category:  Mean (sd) | | | | | | | |
| --- | --- | --- | --- | --- | --- | --- | --- | --- |
|  | **GOSE 1:**  **Death** | **GOSE 2:**  **Vegetative state** | **GOSE 3:**  **Lower severe disability** | **GOSE 4:**  **Upper severe disability** | **GOSE 5:**  **Lower moderate disability** | **GOSE 6:**  **Upper moderate disability** | **GOSE 7:**  **Lower good recovery** | **GOSE 8:**  **Upper good recovery** |
| **n=** | - | 6 | 616 | 284 | 498 | 724 | 564 | 745 |
| **United Kingdom** | 0  (-) | -0.177  (0.19) | 0.325  (0.35) | 0.505  (0.31) | 0.586  (0.30) | 0.735  (0.22) | 0.838  (0.18) | 0.937  (0.12) |
| **Denmark** | 0  (-) | -0.048  (0.20) | 0.447  (0.30) | 0.582  (0.23) | 0.644  (0.23) | 0.758  (0.16) | 0.849  (0.15) | 0.938  (0.11) |
| **Germany** | 0  (-) | 0.327  (0.14) | 0.501  (0.30) | 0.670  (0.27) | 0.741  (0.26) | 0.859  (0.17) | 0.916  (0.14) | 0.970  (0.08) |
| **Netherlands** | 0  (-) | 0.125  (0.18) | 0.432  (0.29) | 0.555  (0.28) | 0.614  (0.28) | 0.754  (0.20) | 0.854  (0.17) | 0.944  (0.11) |
| **Spain** | 0  (-) | -0.397  (0.13) | 0.269  (0.40) | 0.525  (0.30) | 0.634  (0.28) | 0.776  (0.20) | 0.870  (0.16) | 0.952  (0.10) |
| **Japan** | 0  (-) | 0.072  (0.10) | 0.493  (0.21) | 0.605  (0.15) | 0.666  (0.14) | 0.741  (0.138) | 0.828  (0.15) | 0.926  (0.12) |
| **United States** | 0  (-) | 0.092  (0.10) | 0.496  (0.25) | 0.634  (0.20) | 0.696  (0.20) | 0.794  (0.14) | 0.870  (0.13) | 0.946  (0.10) |
| **Zimbabwe** | 0  (-) | 0.184  (0.150 | 0.522  (0.21) | 0.641  (0.17) | 0.707  (0.17) | 0.792  (0.13) | 0.866  (0.13) | 0.945  (0.10) |
| **Friedmann’s test** | - | <0.001 | <0.001 | <0.001 | <0.001 | <0.001 | <0.001 | <0.001 |

*Predictive mixture models for HSPWs for extended GOS categories*

The preferred model predicting EQ5D index scores from extended GOS category and age, chosen on the basis of favourability of goodness of fit statistics (Table 11), included 4 latent classes. Age and extended GOS category demonstrated a statistically significant effect on the probability of latent class membership and the distribution of mean EQ5D scores within each component. In and out of sample prediction of EQ-5D at 12 and 24 months post-injury also showed excellent agreement between observed and predicted values as shown by the cumulative distribution functions in Figure 2. However, there was again under-prediction of EQ-5D at 6 months for those patients with little or no functional disability (Figure 2).

The most favourable detailed model with the extended GOS, including the additional covariates of age, gender, co-morbidity and extra-cranial injury, also used 4 latent classes. Each covariate had a statistically significant association with the EQ-5D distribution within each component. Age, extended GOS and extra-cranial injury significantly predicted the probability of latent class membership. A similar pattern of excellent in-sample (12 months) and out of sample prediction at 24 months post-injury was evident but under-prediction of high EQ-5Ds at 6 months post injury was again observed (Figure 2).

Coefficients and co-variance matrices are available from the author on request. A stata do file allowing calculation of mean EQ-5D with 95% confidence intervals for a given extended GOS and covariates is supplied as an additional file.

**Table 11.** Goodness of fit metrics for simple and detailed models predicting EQ-5D from basic GOS category

| **Simple model: Extended GOS and age** | | | | | |
| --- | --- | --- | --- | --- | --- |
| Number of latent classes | AIC | BIC | Mean error | MAE | RMSE |
| 1 | 1922.622 | 1977.909 | 0.0059 | 0.1864 | 0.2486 |
| 2 | 742.7135 | **871.7151** | -0.0007 | 0.1831 | 0.2470 |
| 3 | 686.5773 | 883.1512 | -0.0005 | 0.1827 | 0.2469 |
| 4 | **638.1157** | 908.4049 | **-0.0001** | **0.1825** | **0.2463** |

AIC: Akaike information criterion; BIC: Bayesian information criterion; MAE: Mean absolute error; RMSE: Root mean squared error. Most favourable result for each metric of goodness of fit highlighted in bold text.

| **Simple model: Extended GOS, age** | **Detailed model: Extended GOS, age, gender, comorbidity, extra-cranial injury** |
| --- | --- |
| Within sample prediction : EQ-5D at 12 months post-injury | |
| 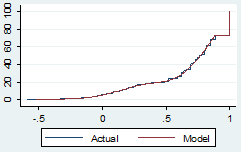 | 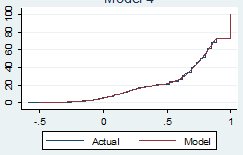 |
| Out of sample prediction: EQ-5D at 6 months post-injury | |
| 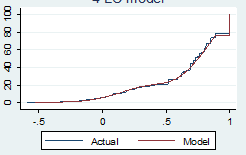 | 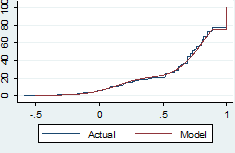 |
| Out of sample prediction: EQ-5D at 24 months post-injury | |
| 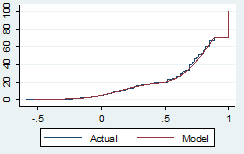 | 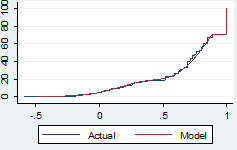 |

**Figure 2. Cumulative distribution functions for observed versus predicted EQ-5D values from extended GOS predictive models.** x-axis denotes EQ-5D; y-axis displays cumulative percentage of cases.

**references**

1. Excellence NIoHaC. Guide to the methods of technology appraisal 2013 In: Excellence NIoHaC, ed. London: NICE, 2013.

2. Cabase\s JME, Gaminde IE. EQ-5D : an instrument to value health from the EuroQol Group : 17th plenary meeting of the EuroQol Group. Universidad Pu\0301blica de Navarra, 2000.

3. Szende A, Oppe M, Devlin NJ. EQ-5D value sets : inventory, comparative review and user guide. Dordrecht ; [London]: Springer, 2007.

4. Dolan P, Gudex C, Kind P. A social tariff for EuroQol : results from a UK general population survey. University of York, Centre for Health Economics, 1995.

5. Herdman M, Gudex C, Lloyd A, et al. Development and preliminary testing of the new five-level version of EQ-5D (EQ-5D-5L). Quality of life research : an international journal of quality of life aspects of treatment, care and rehabilitation. 2011; 20: 1727-36.

6. Brazier J. Measuring and valuing health benefits for economic evaluation. Oxford ; New York: Oxford University Press, 2007.

7. Weinstein MC, Torrance G, McGuire A. QALYs: the basics. Value in health : the journal of the International Society for Pharmacoeconomics and Outcomes Research. 2009; 12 Suppl 1: S5-9.

8. K. T. What are health utilities? London: Hayward Medical Communications., 2009.

9. Drummond MF. Methods for the economic evaluation of health care programmes. 3rd ed. ed. Oxford: Oxford University Press, 2005.

10. Papaioannou D BJ, Paisley S. NICE DSU Technical Support Document 9:The identification, review and synthesis of health state utility values from the literature. . In: Excellence NIoHaC, ed., NICE. London, 2011.

11. Torrance GW. Measurement of health state utilities for economic appraisal. Journal of health economics. 1986; 5: 1-30.

12. Morimoto T, Fukui T. Utilities measured by rating scale, time trade-off, and standard gamble: review and reference for health care professionals. Journal of epidemiology / Japan Epidemiological Association. 2002; 12: 160-78.

13. Higgins JPT, Altman DG, Gotzsche PC, et al. The Cochrane Collaboration's tool for assessing risk of bias in randomised trials. Br Med J. 2011; 343.

14. Hernandez Alava M, Wailoo AJ, Ara R. Tails from the peak district: adjusted limited dependent variable mixture models of EQ-5D questionnaire health state utility values. Value in health : the journal of the International Society for Pharmacoeconomics and Outcomes Research. 2012; 15: 550-61.

15. Hernandez Alava M, Wailoo A, Wolfe F, et al. A Comparison of Direct and Indirect Methods for the Estimation of Health Utilities from Clinical Outcomes. Medical decision making : an international journal of the Society for Medical Decision Making. 2013.

16. Longworth L, Rowen D. Mapping to obtain EQ-5D utility values for use in NICE health technology assessments. Value in health : the journal of the International Society for Pharmacoeconomics and Outcomes Research. 2013; 16: 202-10.

17. Dakin H. Review of studies mapping from quality of life or clinical measures to EQ-5D: an online database. Health Qual Life Outcomes. 2013; 11: 151.

18. McLachlan GJ, Peel D. Finite mixture models. New York ; Chichester: Wiley, 2000.

19. Schlattmann P. Medical applications of finite mixture models. Berlin: Springer, 2009.

20. Steyerberg EW. Clinical prediction models : a practical approach to development, validation, and updating. New York ; London: Springer, 2009.

21. Hernandez-Alava MW, A. ALDVMM: A command for fitting Adjusted Limited Dependent Variable Mixture Models to EQ-5D. The Stata Journal. 2014: In press.

22. Cotton BA, Kao LS, Kozar R, et al. Cost-utility analysis of levetiracetam and phenytoin for posttraumatic seizure prophylaxis. The Journal of trauma. 2011; 71: 375-9.

23. Pandor A, Goodacre S, Harnan S, et al. Diagnostic management strategies for adults and children with minor head injury: a systematic review and an economic evaluation. Health technology assessment (Winchester, England). 2011; 15: 1-202.

24. Smits M, Dippel DW, Nederkoorn PJ, et al. Minor head injury: CT-based strategies for management--a cost-effectiveness analysis. Radiology. 2010; 254: 532-40.

25. Stein SC, Burnett MG, Glick HA. Indications for CT scanning in mild traumatic brain injury: A cost-effectiveness study. The Journal of trauma. 2006; 61: 558-66.

26. Stein SC, Fabbri A, Servadei F. Routine serial computed tomographic scans in mild traumatic brain injury: when are they cost-effective? The Journal of trauma. 2008; 65: 66-72.

27. Whitmore RG, Thawani JP, Grady MS, et al. Is aggressive treatment of traumatic brain injury cost-effective? Journal of neurosurgery. 2012; 116: 1106-13.

28. Alali AS, Burton K, Fowler RA, et al. Economic Evaluations in the Diagnosis and Management of Traumatic Brain Injury: A Systematic Review and Analysis of Quality. Value in health : the journal of the International Society for Pharmacoeconomics and Outcomes Research. 2015; 18: 721-34.

29. Eliason SR. Maximum likelihood estimation : logic and practice. Newbury Park, Calif.: Sage, 1993.

30. Laarhoven PJMv, Aarts EHL. Simulated annealing : theory and applications. Dordrecht ; Lancaster: Reidel, 1987.

31. Kosty J, Macyszyn L, Lai K, et al. Relating quality of life to Glasgow outcome scale health states. J Neurotrauma. 2012; 29: 1322-7.

32. Smits M, Hunink MG, van Rijssel DA, et al. Outcome after complicated minor head injury. AJNR American journal of neuroradiology. 2008; 29: 506-13.

33. Dijkers MP. Quality of life after traumatic brain injury: a review of research approaches and findings. Archives of physical medicine and rehabilitation. 2004; 85: S21-35.

34. Tsauo JY, Hwang JS, Chiu WT, et al. Estimation of expected utility gained from the helmet law in Taiwan by quality-adjusted survival time. Accident; analysis and prevention. 1999; 31: 253-63.

35. Aoki N, Kitahara T, Fukui T, et al. Management of unruptured intracranial aneurysm in Japan: a Markovian decision analysis with utility measurements based on the Glasgow Outcome Scale. Medical decision making : an international journal of the Society for Medical Decision Making. 1998; 18: 357-64.
